# Identification of molecular sub-networks associated with cell survival in a chronically SIVmac-infected human CD4+ T cell line

**DOI:** 10.1186/1743-422X-11-152

**Published:** 2014-08-27

**Authors:** Feng Q He, Ulrike Sauermann, Christiane Beer, Silke Winkelmann, Zheng Yu, Sieghart Sopper, An-Ping Zeng, Manfred Wirth

**Affiliations:** Group Systems Biology, Helmholtz Centre for Infection Research (HZI), Inhoffenstr. 7, D-38124 Braunschweig, Germany; Infection models, German Primate Centre DPZ, Kellnerweg 4, D-37077 Göttingen, Germany; Epigenetic Regulation Mechanisms, Helmholtz Centre for Infection Research (HZI), Inhoffenstr. 7, 38124 Braunschweig, Germany; Tumor Immunology Lab, Hematology and Oncology, Medical University Innsbruck, Innsbruck, Austria; Tyrolean Cancer Research Institute, Innsbruck, Austria; Institute of Bioprocess and Biosystems Engineering, Hamburg University of Technology, Dennickestr. 15, D-21073 Hamburg, Germany; Department of Molecular Biology, Aarhus University, C.F. Mollers Alle 130, Aarhus, Denmark; Luxembourg Centre for Systems Biomedicine (LCSB), University of Luxembourg, 7, Avenue des Hauts Fourneaux, L-4362 Esch-sur-Alzette, Luxembourg

**Keywords:** Human T cell line, Chronic SIV/HIV infection, Virus-host-interaction, Transcriptome, Network analysis, Caveolin-1, CD4, Key gene prediction, Systems biology

## Abstract

**Background:**

The deciphering of cellular networks to determine susceptibility to infection by HIV or the related simian immunodeficiency virus (SIV) is a major challenge in infection biology.

**Results:**

Here, we have compared gene expression profiles of a human CD4^+^ T cell line at 24 h after infection with a cell line of the same origin permanently releasing SIV_mac_. A new knowledge-based-network approach (*Inter-Chain-Finder, ICF*) has been used to identify sub-networks associated with cell survival of a chronically SIV-infected T cell line. Notably, the method can identify not only differentially expressed key hub genes but also non-differentially expressed, critical, ‘hidden’ regulators. Six out of the 13 predicted major hidden key regulators were among the landscape of proteins known to interact with HIV. Several sub-networks were dysregulated upon chronic infection with SIV. Most prominently, factors reported to be engaged in early stages of acute viral infection were affected, e.g. entry, integration and provirus transcription and other cellular responses such as apoptosis and proliferation were modulated. For experimental validation of the gene expression analyses and computational predictions, individual pathways/sub-networks and significantly altered key regulators were investigated further. We showed that the expression of caveolin-1 (Cav-1), the top hub in the affected protein-protein interaction network, was significantly upregulated in chronically SIV-infected CD4^+^ T cells. Cav-1 is the main determinant of caveolae and a central component of several signal transduction pathways. Furthermore, CD4 downregulation and modulation of the expression of alternate and co-receptors as well as pathways associated with viral integration into the genome were also observed in these cells. Putatively, these modifications interfere with re-infection and the early replication cycle and inhibit cell death provoked by syncytia formation and bystander apoptosis.

**Conclusions:**

Thus, by using the novel approach for network analysis, *ICF*, we predict that in the T cell line chronically infected with SIV, cellular processes that are known to be crucial for early phases of HIV/SIV replication are altered and cellular responses that result in cell death are modulated. These modifications presumably contribute to cell survival despite chronic infection.

**Electronic supplementary material:**

The online version of this article (doi:10.1186/1743-422X-11-152) contains supplementary material, which is available to authorized users.

## Background

SIV (simian immunodeficiency virus)-infected macaques represent the most accepted model for HIV infection. Therefore, the investigation of SIV/host interactions gives valuable insights into cellular strategies against viral invasions and illuminates viral strategies to counteract cellular defense [1]. Three different types of HIV/SIV infections can be distinguished. Acute infection is characterized by virus replication and subsequent lysis of CD4^+^ T cells. In a latent infection, virus production is extremely low or repressed, whereas the virus genome is still detectable and viruses reside in a poorly defined cellular reservoir. This type of infection can be observed in elite controllers. A pattern of infection mainly observed *in vitro* refers to a chronic infection that is characterized by a permanent virus production in cells that are not harmed by virus replication. Although a considerable number of factors have been identified as interfering with the HIV replication cycle in T cells and although factors that give rise to the survival of HIV-1-infected macrophages have been reported, the determinants of the resistance of certain patients to HIV-1 infection are not fully understood [2–8]. Thus, the permanently SIV/HIV CD4^+^ producing T cell lines are valuable models for studying survival mechanisms in cells that represent primary targets of HIV-1/SIV infection. The monitoring of changes in gene expression on a genome scale is a powerful tool for examining transcriptional programs involved in virus pathogenesis. To date, several investigations using gene expression profiling for understanding HIV/SIV host interaction have been reported [1, 9–15]. In order to obtain greater insights into the genetic networks, main regulators and mechanisms associated with cell survival in a chronic infection, we have compared the cellular responses to acute and chronic types of SIV-infection of a human CD4^+^ T cell line. A human T cell line was chosen for scientific reasons, because little is known of the gene expression pattern in SIV-infected human T cells, and for technical reasons, because of the unique availability of this specific cell line. Furthermore, the current version of the computational approach (*Inter-Chain-Finder, ICF*) is based on molecular interactions in human cells.

To identify molecules at a systemic level that might govern cell survival despite chronic SIV infection of the human CD4^+^ T cell line, we have applied a conventional pathway analysis of cDNA microarray data. Furthermore, we have implemented a new strategy for knowledge-based network analysis able to localize significantly affected sub-networks and non-differentially transcribed (‘hidden’) key transcription factors. Identification of the latter is very challenging but essential since many regulators are only post-transcriptionally activated/inhibited and such alterations are not recorded by standard microarray analysis. Following the network-based analysis, selected targets have been subsequently studied by experimental investigations. Our results indicate that early steps in viral multiplication (entry, integration, transcription) are preferentially affected in chronically SIV-infected T cells; this together with other factors might contribute to the phenotypes observed.

## Results and discussion

### Characterization of a lytically SIV_mac251/H32_ -infected human CD4^+^ T cell line and a model CD4^+^ T cell line permanently releasing SIV with respect to virus count and proviral state

To identify a survival network in the chronic type of infection, we compared the global gene expression of the permanently SIV-releasing CD4^+^ human T cell line C8166 (C8166-P) with the gene expression pattern of acutely infected and mock-infected cells [16]. An early time-point (24 h) after acute infection was chosen to study the appearance of cellular responses, extensive replication leading to cell damage not having yet occurred.

To characterize the individual courses of SIV infection, we compared a combination of various parameters, such as virus copy numbers, percentage of infected cells, the virus particle numbers released and the chromosomal status of the provirus. Determination of viral DNA copy numbers by polymerase chain reaction (PCR) revealed that 0.3 copies of SIV DNA per cell were found on average in the acute SIV infection, whereas 2–3 copies of the SIV DNA were present in permanently SIV-releasing T cells (Table 1). As infected cells can harbour up to 8 proviruses, and the cell line is in permanent contact with infectious virus, it is speculated that the permanently SIV-releasing cell line resists some degree of re-infection [17]. An indirect immunoperoxidase assay was used to determine the percentage of infected cells in acute and chronic infection. In acute infection, about 25% of cells were positive for SIV staining, whereas all of the chronically infected T cells expressed viral proteins. Furthermore, the numbers of viruses released from the two experimental settings were determined by PCR analysis. The population of acutely infected cells is heterogeneous. Thus, gene expression analysis may have been affected through a change of the transcriptional profile by contact of CD4 or CCR5 with Env on virions on infected and uninfected cells [18]. To account for that fact and to achieve a kind of standardization we introduced a ‘multiplicity of contact’ concept into our experimental settings. For that purpose, we determined viral particle numbers (see M&M Infection and RNA isolation for details) and took care, that the number of contacts with virions and cells was similar for both cell lines.

**Table 1:** **Comparison of viral load in lytic versus chronic infection of CD4** ^**+**^ **T cell line C8166**

SIV _mac251_/C8166	Lytic infection	Chronic infection
Percentage of infected cells^1^	25%	100%
Copy number^2^	0.3	2-3
Viruses in supernatant^3^	3	300
Provirus integration^4^	18%	n.d.^5^

^1^As determined by indirect immune peroxidase assay.

^2^Copies per SIV DNA as determined by proviral specific PCR.

^3^Particles per cell at one day after infection or medium exchange by means of PCR analysis.

^4^Halo-FISH analysis, integration into transcriptionally active matrix regions.

^5^ n.d. not detectable by Halo-FISH analysis.

**Figure 1:**
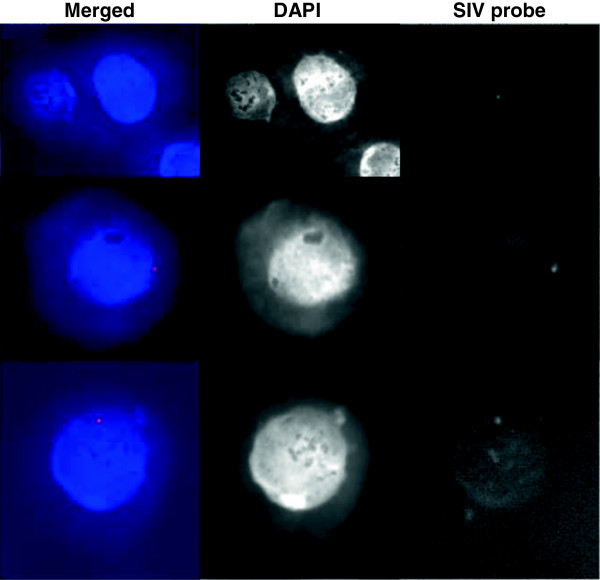
**Halo**-**FISH analysis of acutely SIV-infected C8166 T cells.** Integration of SIV into the genomic DNA of the C8166 T cell line. Three acutely SIV-infected cells were shown in three different rows. Halo-FISH analyses of cells 1 d after acute infection was performed by using a SIV gag-env DNA probe. Nuclei were visualized by DAPI staining. Single integration events of SIV proviral DNA (SIV probe, white spots; Merged with DAPI staining, red spots) into the nuclear matrix at regions of high transcriptional activity (blue sphere) are clearly visible. No integration into the associated HALO (regions of low transcriptional activity surrounding the nuclear matrix) was observed.

Infection experiments also revealed a low number of viral particles (3 per cell on average) in the supernatant of acutely infected T cells after day 1 which is consistent with the notion that a full SIV-replication round is not completed at that time. On the contrary, chronically SIV-infected T cells released 300 SIV particles per cell on average. Obviously, the released viruses do not harm neighbouring T cells, albeit they are highly infectious and replicate to a similar extent as SIVmac239 and SIVmac251 in naïve C8166 cells and monkey lymphocytes (Additional file 1: Figure S1). Last, the chromosomal status of provirus in acute infection was determined by Halo-FISH (fluorescence *in situ* hybridization) analysis (Figure 1). In addition to copy number determination, this method differentiates between integration into loop and matrix-attached regions of the chromosome. By using the probe pGX10-SIV-GE, which contains Gag and Env coding regions of SIVmac251, Halo-FISH showed that about 18% of the acutely infected cells harboured provirus that was integrated predominantly into the matrix regions (transcriptionally active domains), but not into loop-regions (transcriptionally silent domains) (Figure 1). Thus, the different techniques for estimating the percentage of acutely infected cells or viral DNA copy number were in relative good agreement. Interestingly, despite various modifications in salt extraction, the Halo-Fish method failed to give results regarding the chromosomal integration status of SIV in chronically infected T cells. The reason for this is not clear but indicates that the inner milieu of these cells has changed dramatically.

### Principle, features and generality of the network strategy

**Figure 2:**
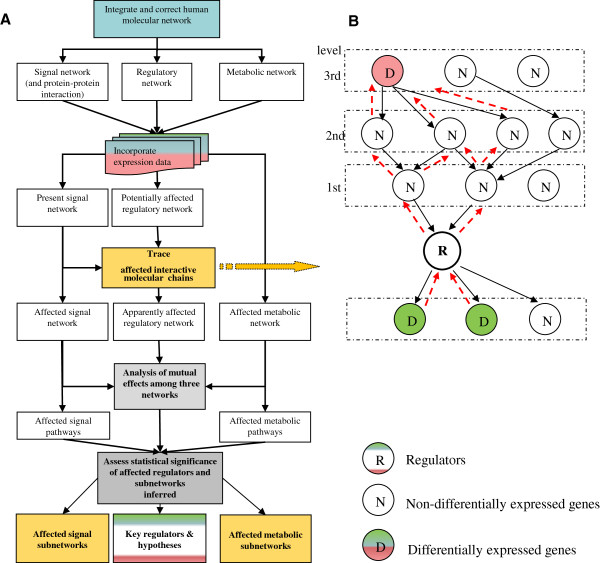
**Strategy and principle of** ***Inter-Chain-Finder (ICF).*** **A**. Strategy of deciphering the response of model organisms to conditional changes on the genome scale by an integrative approach based on an integrated network. First, an integrated network is constructed. Gene-profiling data are subsequently used to identify affected interactions in the integrated network at the molecular levels. The *ICF* then traces back all affected interactive molecular chains (IMCs) and identifies non-differentially (NDiff) and differentially expressed (Diff) key regulators. Finally, regulators and the affected sub-networks are displayed by Cytoscape. The steps in bold are the core steps. The key steps are explained in detail in the Materials and methods. **B**. Demonstration of the tracing approach for possible, significantly affected IMCs mapping regulators, their upstream effectors and downstream targets. Black arrow represents the direction of IMCs in the available signal network; red dashed arrow represents the direction of chain tracing. R: Regulators (white, NDiff, green/red, Diff); D: Diff genes, red: upregulated, green: downregulated; N: NDiff genes, white.

An essential limitation of microarray-based techniques is that post-transcriptional modifications that regulate cellular processes by activation or inhibition cannot be detected at all. To partially compensate this lack, we applied a novel strategy termed *Inter-Chain-Finder* (*ICF*) which is able to identify upstream regulators whose transcriptional change and/or predicted change of transcriptional regulatory activity can drive differential expression of target genes. The *ICF* can uncover significantly affected interactive molecular chains (IMCs) that might affect the activities of non-differentially-expressed (NDiff) transcription factors. A representation of the new strategy is depicted in Figure 2 (for details, see Materials and methods). First, an integrated human molecular network is constructed by including information on transcriptional regulations, signalling transduction interactions (or protein-protein interactions) and metabolic reactions. Then, the expression data are used to reveal changes in the integrated network at various molecular levels. The *ICF* subsequently systemically traces and identifies all the possibly significantly affected IMCs that might modulate the activities of NDiff regulators (Figure 2 and Additional file 2: Figure S3). The essential hypothesis behind *ICF* is that if both upstream and downstream factors of a given non-differentially transcribed regulator are significantly affected, the activity of the given NDiff regulator is also most likely affected (for details, see Materials and methods, the scheme in Figure 2 and the example in Additional file 3: Figure S4).

In contrast to the other available approaches which can mainly identify differentially expressed (Diff) genes or Diff networks or pathways, the *ICF* not only uncovers the signal transduction, metabolic and regulatory sub-networks disturbed at the transcriptional level upon the conditional changes, but also reveals NDiff ‘hidden’ regulators and explains how the Diff genes are regulated. This is achieved by tracing back affected signaling transduction chains. The capability of being able to infer and interpret NDiff potential ‘hidden’ key regulators has partially compensated the essential limitation of microarray-based techniques in which post-transcriptional modifications cannot be detected at all. Furthermore, we identify and rank affected sub-networks based on calculation of the number of affected interactions rather than the number of Diff genes and with no requirement of the differential expression for each individual gene in the affected interactions (see Materials and methods). Since all biological processes are exerted through molecular interactions rather than independent genes/proteins, we believe that the calculation based on interactions is more biologically relevant than that based on individual genes by other approaches.

The revelation of such sub-networks and potential ‘hidden’ key regulators is the original aspect of this work compared with other methods of network-based analysis, which are, for example, applied for the analysis of systemic inflammation in humans [19], specific inflammatory diseases [20, 21] or lung cancer [22]. Although the method used for lung cancer analysis [22] previously noted “hidden” regulators, the strategy did not trace significantly affected IMCs in order to identify those regulators. Finally, *ICF* can be generally used to uncover molecular responsive networks of various organisms provided a corresponding integrated molecular network of the respective organism and sufficient knowledge of its molecular interactions are available.

### Permanently SIV-releasing T cells are characterized by activation of multiple survival sub-networks that interfere with viral replication cycle

To investigate the way that the type of infection affects gene expression at the transcriptional level, genome-wide transcription was measured in chronically, acutely infected and non-infected control CD4^+^ T cells (Affymetrix HG_U133A microarray, measured by Dr. Geffers, Microarray platform, HZI). Two independent experiments were done 6 months apart and cells from different passages were used to minimize problems related to the genetic drifting of the cell lines. Data from chronically and acutely infected cells and mock-infection were compared after threshold levels had been defined by standard methods (see Materials and methods). Upon chronic infection, 190 genes were upregulated and 139 downregulated compared with control cells (Additional file 4). Acute infection resulted in the upregulation of only 22 genes and the downregulation of 9 genes.

**Figure 3:**
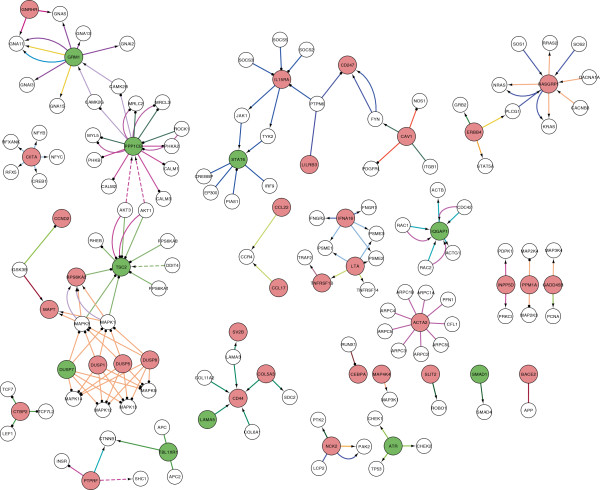
**Main parts of signal transduction network significantly perturbed in chronically infected SIV C8166 CD4+ T cells.** The different colours of interactions correspond to different pathways as annotated by KEGG database (for details see the integrated database). The black arrow represents activation (detailed relationship of interactions can be obtained in the database from authors on request); the black circle represents inhibition; no edge end represents the undirected relationship PPI of the two proteins; the dashed line represents an indirect relationship. Red color, significantly increased (see Materials and methods); green, significantly decreased; white, NDiff.

**Figure 4:**
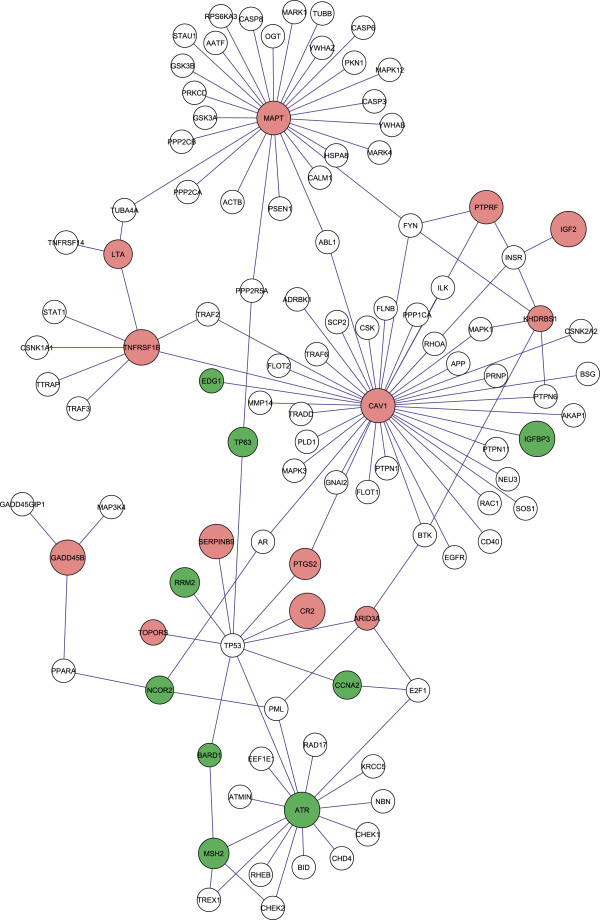
**Parts of the protein-protein network of CD4+ T cells significantly perturbed only during the chronic infection of SIV.** A zoom-in of the protein-protein network depicted in Additional file 5: Figure S2 is shown. Red colour, significantly increased; green, significantly decreased; white, non-differentially expressed. Note the binding partners of Cav-1 and the linkages of Cav-1 to several other sub-networks, e.g. insulin module (IGF2, IGFBP3) and p53 (TP53) subnetworks.

Affected biochemical and regulatory pathways were identified initially by visual inspection, the GO-viewer software (Guido Diettrich, HZI) and the DAVID annotation program [23] with emphasis on Diff genes and gene families. Sub-networks and several presumed key regulators, which are, however, not necessarily differentially expressed, were identified by the new network-based method *ICF* (for details of the strategy, see Materials and methods and the scheme in Figure 2). A selection of affected sub-networks is depicted in Figures 3 and 4 and in Additional file 5: Figure S2.

**Figure 5:**
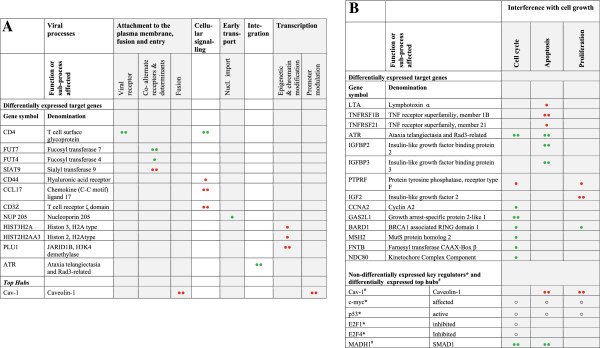
**Cellular responses in chronically SIV-infected T cells and reported involvement in stages in acute SIV/HIV-1 infection of susceptible cell types: putative contributions to cell survival.** The perturbation of cellular processes known to be important for the early viral replication cycle **(A)** and activity of cell growth genes **(B)** is predicted to confer survival of chronically SIV-infected T cells. Affected genes are listed from microarrays of SIV-resistant T cells and are associated with their reported roles in the viral replication cycle. Alterations in mRNA levels were not observed in acutely infected cells at 24 h after infection. Abbreviations and symbols: * or ^#^ inferred by the *ICF*. Differentially (green circle or red circle) and non-differentially (○) expressed genes, suggested activity and regulator status. Cellular response 2- to 5-fold decrease (green circle) or increase (red circle); more than 5-fold decrease (two green circles) or increase (two red circles); (○) no significant change in mRNA level.

In the chronically SIV-infected cells, genes controlling cell cycle and apoptosis were affected similar to results from chronically infected HIV T cell lines. Most strikingly, mainly sub-networks and gene families were affected in the chronically SIV-infected T cell line that are involved in early steps of an acute infection in the viral replication cycle, rather than in the late phase, such as viral entry, integration and early responses to viral infection. In the following, a selection of several sub-networks linked to these responses is presented (for a functional compilation of selected affected genes, see Figure 5).

### Loss of CD4 gene expression and putative impairment of alternate routes of virus attachment and entry

#### Downregulation of CD4

**Figure 6:**
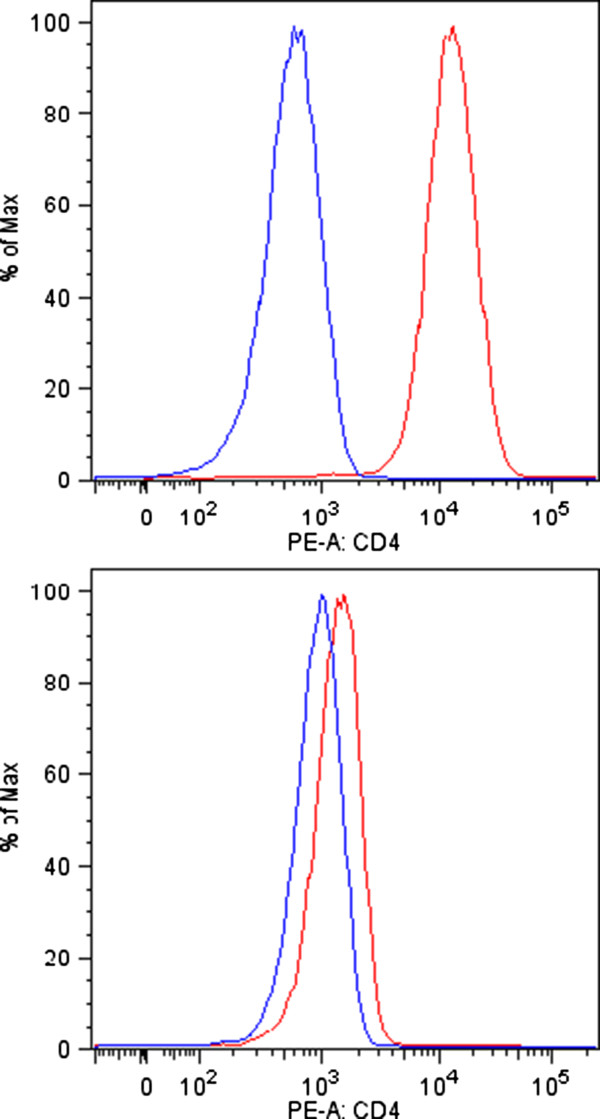
**CD4 surface expression in SIV-infected C8166 T cell line determined by FACS analysis.** CD4 surface expression on uninfected (upper panel) and chronically infected (lower panel) C-8166 cells as determined by flow cytometry. Blue lines depict unstained control samples, red lines staining with an antibody directed against CD4. Chronically infected cells display a slightly higher auto-fluorescence and only a little CD4 expression compared with uninfected cells.

SIV and HIV-1 entry are multi-step processes and the mechanism of entry is cell-dependent. CD4 represents the most important cellular receptor for HIV-1 and SIV and is involved in syncytium formation in infected cells. The microarray experiments, real-time RT-PCR (reverse transcription with PCR) and FACS (fluorescence-activated cell sorting) analysis revealed that CD4 mRNA levels and cell surface expression were significantly reduced in chronically SIV-infected T cells (Figures 5, 6 and Table 2). On average, the real-time RT-PCR results showed a 14- fold decrease in CD4 RNA abundance in the chronically SIV-infected CD4^+^ T cell line compared with mock-infected cells (Table 2). The CD4 protein was only barely detectable by FACS analysis in the permanently SIV-producing C8166 cells (Figure 6). Thus, in addition to the known mechanisms of CD4 protein reduction by Env, Vpu and Nef [24–28], CD4 expression appeared to be further repressed in the permanently SIV-producing cell at the transcriptional level. This is interesting, as transcriptional silencing might serve for complete CD4 removal from the surface.

**Table 2:** **Real-time RT-PCR analysis of suspected key components of the response to chronic SIV-infection**

Gene	Microarray		RT-PCR	
	Acute ^1^	Chronic ^1^	Acute	Chronic
Cav-1	1	4.82	1	4
CCL3	1	0.7	1	1
CCR5	1	1.4	n. d.^2^	>20
CD3Z	1	11.68	1	13
CD4	1	0.12	1.8	0.06
CD44	1	2.69	1.35	11
Fut-7	1	0.12	1.25	0.095
IL2-RA	1	0.59	1.25	0.29

^1^RNA from acutely or chronically SIV_mac251_-infected or mock-infected C8166 T cells was used for the experiments. Values represent the ratios between signals (average fold change) derived from infected and mock-infected cells and are derived from at least 2 independent experiments.

^2^not detectable.

On the contrary, CCR5, the co-receptor used by SIV, was hardly detectable in acute infection but was considerably increased in chronic infection (Table 2). Chemokine mRNAs of CCL3 (Table 2) or CCL5 (not shown) seem not to be affected.

Thus, CD4 loss should result in reduced infection, syncytium formation, and Env-mediated apoptosis of chronically infected cells considerably contributing to their survival. In support of our assumption, down-modulation of plasma membrane CD4 has previously been shown to result in resistance to superinfection mediated by SIV Nef protein and linked to the interference with re-infection in chronically HIV-1 infected T cells [29–31]. Reduction of CD4 cell surface expression has also been reported to result in particles with fewer CD4 and more Env molecules, which probably eases their release [6, 7].

#### Impairment of alternate routes of infection

Infection by HIV/SIV is known to be facilitated by alternate receptors or alternate routes, if the primary viral receptor/co-receptor is missing or blocked [32–36]. In many cases, the HIV-interacting counterparts are lectins, which are glycoproteins that interact with the highly glycosylated lentiviral envelopes.

Strikingly, the glycosylation potential of permanently SIV-releasing T cells might be altered, as the gene expression of several modifying glycosyltransferases was decreased. For example, the gene expression of fucosyltransferase IV and VII (FUT4, FUT7), which add fucose to glycans at the α-1,3 position, was diminished as determined in microarray and real-time PCR experiments (Figure 5A, Table 2). Branched fucose oligosaccharides play a role in attachment to the high mannose receptor DC-SIGN [37, 38]. DC-SIGN, a C-type lectin abundant in dendritic cells, acts as an HIV receptor by binding Env in a glycosylation-dependent manner [39, 40]. Moreover, the expression of a GlcNac-0-6 sulfotransferase (CHST7) was increased in chronically SIV infected cells (data not shown) and this might have an effect on HIV-binding to cells as the type and amount of sulfation of Env carbohydrate residues have been shown to affect Env affinity to DC-SIGN, CCR5 or syndecans [37, 38, 41]. Furthermore, sialyltransferase 9 (SIAT9, also termed ST3Gal5a) expression was considerably upregulated in the chronically SIV-infected T cells (Figure 5A). SIAT-9 is responsible for the synthesis of GM3, a glycosphingolipid. Interestingly, GM3 has been identified as an attachment factor for HIV-1, thereby facilitating HIV infection under physiological conditions, but clearly inhibiting HIV infection upon overexpression [42]. Finally, we have found that syndecan 4 (SDC4) expression tended to downregulation in chronically SIV-infected T cells, suggesting that another bypass in infection might be compromised. Syndecans, including syndecan 4, have been demonstrated to be able to function as *cis* and *trans* HIV receptors via the binding of HIV-1 gp120 to the heparan sulfate chains of syndecan. Syndecans can promote HIV infection in the absence of CD4 in macrophage and epithelial cells. Thus, syndecans perform multiple roles as they capture, protect and transmit HIV to T cells [34, 43].

Taken together, the results suggest that a modified carbohydrate moiety in chronically infected T cells might impair the attachment of SIV and also restrict alternate routes of infection.

### Significantly affected sub-networks involving caveolin-1

**Figure 7:**
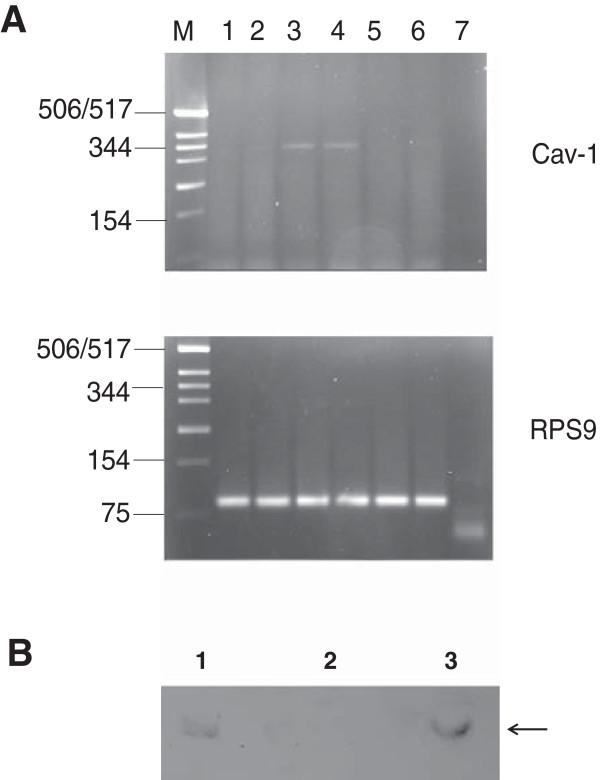
**Caveolin-1 expression is induced in chronically infected C8166 T cells. A**: Caveolin-1 mRNA expression is induced in human T cell chronically infected by SIV. Products from LightCycler PCR from mRNA isolated from infected and mock-infected T cells are shown. Specific exon-spanning primers were used to amplify Cav-1 or ribosomal protein 9 (RPS9) for normalization. Lanes 1, 2 acute infection; lanes 3, 4 chronically SIV-infected T cells; lanes 5, 6 mock-infected cells; lane 7, water control. The expected products of 318 bp (top Cav-1 lanes 3, 4) and 95 bp (bottom RPS9, lanes 1–6) were identified in 2% agarose gels. **B**: Western blot detection of caveolin-1 in SIV_mac251_ H32 C8166-P cells. Equal amounts of cell lysates from human T cell C8166 (lane 2) and from human T cell C8166 chronically infected by SIV (lane 3) were separated by SDS PAGE and Western blot was carried out by using rabbit anti-caveolin-1 antibody as the primary antibody. NIH 3T3 cell lysate (1/50 dilution) was used as the caveolin-1 positive control sample (lane1). Arrow depicts caveolin-1; in lane 1, the two isoforms of caveolin-1 found in NIH 3T3 cells are visible.

By investigating the affected protein-protein network, we could identify the most significantly affected hubs. Additional file 6: Table S1 lists the top 20 upregulated hubs (for the downregulated hubs, see Additional file 7: Table S2). Our results showed that caveolin-1 (Cav-1) was the first among the upregulated hubs (Figures 3, 4, and 5A and B, Additional file 5: Figure S2). In our protein-protein network, we found that 40 out of 81 known interactions with Cav-1 were affected (cumulative binomial distribution, *P-value* is 0, Additional file 6: Table S1). As hubs in the identified network might represent essential genes for physiological or pathological processes [44–47], we considered Cav-1 as a key responsive gene. Microarray results showed that Cav-1 RNA abundance was increased nearly 5-fold in chronically infected T cells. In addition, real-time RT-PCR experiments demonstrated a 4-fold increase in Cav-1 RNA level in SIV C8166 producing cells, whereas the RNA level in acutely infected cells remained unchanged (Table 2). Cav-1 protein expression was verified in Western blots, in which the 22 kD protein was present in chronically infected cells, but absent in the mock-infected control (Figure 7). Thus, caveolin-1 expression was induced in C8166 T cells chronically infected with SIV. This is surprising, as Cav-1 is usually not expressed in CD4^+^ T cell lines [48, 49] and in resting and TCR-stimulated effector CD4+ T cells [44]. Membrane-associated Cav-1 represents the main component of caveolae and functions as a mediator in various signalling processes by establishing signalling-competent complexes by binding through a scaffolding domain within Cav-1. More recent data suggest that Cav-1 also orchestrates T-cell receptor function in CD8^+^ T cells [50]. Furthermore, Cav-1 is involved in intracellular molecule transport and positioning, e.g. it acts as a transporter of intracellular cholesterol and also positions newly synthesized Gag of the γ–retrovirus mouse leukemia virus (MLV) at lipid rafts [51, 52]. Notably, various interactions with matrix proteins of other viral families have been reported, e.g. influenza virus and parainfluenza virus 5 [53–55]. Caveolae have been implicated in SV40 virus entry; however, caveolae participation in the HIV/SIV entry process has not been reported.

Thus, Cav-1 contributes in a structural manner and binds proteins for positioning in or at lipid rafts, transport through the cytoplasm or interference with the function of viral proteins. Furthermore, Cav-1 might orchestrate cellular signalling events leading to cell survival in SIV infection. Various sub-networks, such as the activation of the insulin subnetwork represented by upregulation of (PTPRF, INSR, IGF2) (Figure 4) and downregulation of the inhibitor PPP1CB (protein phosphatase 1, catalytic subunit, beta isoform) of phosphorylase kinase alpha 1 (PHKA1) (Figure 3, Additional file 2: Figure S3 and Additional file 5: Figure S2), in which caveolin-1 is involved [56] have been identified by our network strategy *ICF*. The identification of the insulin module is corroborated by the recent finding, obtained by an RNAi screen, showing that Akt (protein kinase B), another player in the insulin-regulated network, is involved in HIV-1 replication in infected cells, linking viral replication to cellular survival and energy metabolism. Another example comprises the known EGFR binding of Cav-1 and upregulation of CD44 (hyaluronic acid receptor) (Figures 3 and 4, Table 2). CD44 is linked to T cell development and signal transduction leading to proliferation. Furthermore, integrin-fyn interaction, the insulin pathway activation (PTPRF, INSR) (Figure 4) and the involvement in a MAPK pathway resulting in proliferative stimuli (Additional file 2: Figure S3) have been reported.

Caveolin-1 contribution to the survival of chronically SIV-infected T cells is intriguing, but remains speculative and needs confirmation, e.g. by experimental data investigating the influence of SIV infection on CD4+ T cells engineered to express Cav-1. Supporting evidence for a biological relevance of Cav-1 via gp41 binding has been published recently [57–62]. These interactions have been assumed to modulate the viral fusion process or endocytosis. Macrophages do not undergo lysis upon HIV-1 infection and, therefore, are a suspected virus reservoir. Recently, a contribution of Cav-1 to the modulation of bystander apoptosis in HIV-1-infected macrophages has been suggested. Strikingly, in these experiments, Cav-1 restricts virus production and cell death through gp41 binding and inhibition of Env-mediated bystander-apoptosis of non-infected cells [63]. HIV-Tat has been noted as being responsible for the induction of Cav-1 expression, with p53 activation being a necessary step within that pathway (see below, and ref. [64]). Moreover, Cav-1 reduces HIV-1 replication in infected macrophages by interfering with LTR transcription by repressing NF-κB activity, presumably via the acetylation of NF-κB [57, 65]. Furthermore, Cav-1 restores the efflux of cholesterol impaired by viral Nef, thereby interfering with virus budding from rafts and virus infectivity [66, 67]. Hence, Cav-1 is developing as a central molecule, putatively engaged in distinct mechanisms leading to the reduction of infectivity, interference with virus replication and apoptosis in chronic SIV infection of T cells. Our results suggest that Cav-1 contributes to the phenomena observed in chronic SIV/HIV infection of T-lymphocytes, either by its signalling or structural properties.

### Identification of non-differentially expressed key regulators E2F1/4, p53 and Myc, and deregulation of apoptosis, cell cycle and proliferation sub-networks

In general, HIV/SIVinfection induces cell-cycle arrest in the G2/M phase and apoptosis of infected CD4^+^ T cells and adjacent bystander cells. Since chronically SIV-infected C8166 survived the detrimental effects of the infection, the gene expression of the apoptosis and proliferation sub-networks was followed and assessed. Both supportive and inhibitory factors were identified at the transcriptional level (Figures 3, 4 and 5).

Furthermore, using the network strategy *Inter-Chain-Finder (ICF)*, we identified several non-differentially expressed key regulators (p53, E2F1/4, Myc/Max) that play central roles in cellular processes. The mRNA levels of these key regulators were not significantly affected in either SIV-infected cell line. Their involvement in the regulation was inferred by tracing the interacting network (Additional file 2: Figure S3 and Additional file 3: Figure S4). Both the upstream and downstream alterations jointly pointed out the change in activity of those regulators. These findings currently are only indirect evidence. Nevertheless, a recent compilation of data generating a landscape of cellular protein interactions with viruses supports the findings that p53, E2F1 and c-Myc play important roles in the HIV viral replication cycle [68]. In total, 6 out of the 13 predicted ‘hidden’ key regulators listed in Additional file 8: Table S3 were among the landscape of proteins interacting with HIV (Myc, E2F4, RBL2, E2F1, TP53, EGR1) [68, 69]. These candidates have in common that they can regulate cell growth. Obviously, the findings underline the effectiveness of the *ICF* strategy.

p53 (TP53) was predicted by *ICF* as a key regulator in the chronically infected C8166 T cell line, because of the signalling pathways affecting its activities and its effector/target transcriptional pattern (Additional file 2: Figure S3 and Additional file 3: Figure S4). Eight out of the 54 known PDs were affected (*P-value* = 2.11E-5) as identified by the *ICF* (Additional file 8: Table S3). The number of up- or downregulated target genes of p53 was equal (Additional file 3: Figure S4). The results suggested that p53 was activated in the permanently SIV-producing cell line. P53 might lead to the induction of Cav-1 expression, which has been reported for HIV- infected macrophages.

Mainly, p53 functions in apoptosis induced by DNA damage and in cell cycle regulation sustaining G1 and G2 arrest. In general, apoptosis is triggered by external signals via plasma membrane receptors (extrinsic FAS or TNF-α receptor pathway) or within the cell via mitochondria and associated proteins (intrinsic pathway). The initial trigger is transmitted to regulator caspases and followed by executor caspases that finally interfere with cell integrity. P53 activation results in the stimulation of the intrinsic or extrinsic pathway of apoptosis, processes that also can occur in chronically SIV infected T cells because of the predicted p53 activation. Increased Cav-1 levels contribute to apoptosis inhibition [63, 64]. Further experiments are therefore necessary to confirm the p53 status in chronically SIV-infected T cells and to clarify its cellular and viral implications.

The expression of at least 26 genes (Additional file 4) of the apoptosis pathway in mammals was deregulated in the chronically SIV-infected T cell line. We found pro-apoptotic proteins significantly upregulated, such as lymphotoxin alpha (TNF superfamily, member 1) (LTA) and its receptor TNFRSF1B (Figures 3 and 4).

The expression of TRAF-1, which is able to mediate anti-apoptotic signals from TNF-receptors, appeared to be repressed (data not shown). The expression of pro-apoptotic genes, such as caspases 3 and 8, which has been reported to be upregulated by viral Vpr and Tat-proteins, respectively, was not affected.

The expression of pro-survival genes was also observed [70]. Notably, the insulin pathway, which is involved in proliferation, was activated (PTPRF, INSR, IGF2, Figure 4). However, insulin-like growth factor binding protein 3 (IGFBP3), an anti-apoptotic protein, was downregulated in chronically SIV-infected T cells (Figure 4).

Induction of apoptosis of CD4+ T cell as a result of HIV/SIV infection is complex and caused by several mechanisms elicited by viral and cellular proteins [71, 72]. Pro- as well as anti-apoptotic signals were observed upon a chronic course of SIV-infection. However, the net effect of these opposite reactions results in an inhibition of apoptosis.

Furthermore, the *ICF* predicted a change in E2F1/E2F4 activity (Additional file 2: Figures S3 and Additional file 3: Figure S4). Transcription factor E2F1 initiates proliferation and apoptosis. Interestingly, the balance of cell proliferation and apoptosis has recently been shown to be regulated by a subset of E2F1 target genes that are specifically repressed by PI3K/Akt signalling [73]. Proliferation is initiated when E2F has been activated through the phosphorylation of its inhibitor Rb (Retinoblastoma protein) by cyclin D/ CDK4/6 kinase complex [74]. Whereas the genes directing E2F-linked apoptosis were not affected, 8 out of 9 E2F1 targets linked to proliferation (CCNA2, BARD1, KIF11, MSH2, PBK, GAS2L1, FNTB, NDC80) (Additional file 3: Figure S4, Figure 5B) were downregulated in the permanently SIV-releasing cells compared with the control, predicting alterations in cell growth. Of note, whereas upstream IMCs were clearly affected, the P-value for E2F1-affected target genes (also for RBL2 from control samples) indicated a low significance (Additional file 8: Table S3). However, the low significance needs to be interpreted with caution because of the large number of reported PD interactions from high-throughput measurements, which might include a considerable number of false positives. Anyway, the finding that eight out of the nine E2F1 targets were involved in the proliferation process adds high confidence to the importance of E2F1.

CCNA2 (Cyclin A2), FNTB (farnesyl transferase, CAAX box, beta) and NDC80 (kinetochore complex component) promote cell-cycle transitions, whereas the expression of BARD1 (BRCA1 associated RING domain 1), MSH2 (mutS homolog 2, colon cancer, nonpolyposis type 1 (E. coli)), GAS2L1 (growth arrest-specific 2 like 1) is important for cell cycle arrest. Although the eventual effect (promoting or arresting) on cell cycle progression is a tug of war between the two groups of these genes, the downregulation of these genes probably counteracts SIV-induced cell cycle arrest, which has been reported to occur in the G2/M phase and is mediated by the viral Vpr protein [75].

In addition, several genes interacting with E2F1/E2F4 and regulating DNA damage repair (NBN, ATR) were in the disturbed sub-network (Figure 4). ATR, NBN, and CHEK1 are involved in the process of DNA damage-induced checkpoint activation. Furthermore, five genes of 42 genes for the cell cycle checkpoint were found in the ATR (ataxia telangiectasia and Rad3 related) sub-network (binomial distribution probability P-value = 1.27E-6), which were affected in chronically infected C8166, namely ataxia telangiectasia and Rad3 related (ATR), p53 (TP53), nibrin (NBN), pro-myelocytic leukemia (PML) and CHK1 checkpoint homolog (CHEK1) (Figure 4). Although CHEK1 and PML did not show a significant mRNA change, other genes interacting with them in the sub-network were significantly affected in transcription (Figure 4). Those signal transduction or protein-protein interactions were, therefore, possibly affected. Consequently, the related sub-network as a whole might be influenced in executing its functions.

Notably, processes of DNA damage regulation are known to accompany retroviral integration. For instance, PML and integrase interactor 1 (INI1, hSNF5) associate with the HIV-1 integrase-containing pre-integration complexes before they migrate to the nucleus [76]. Furthermore, ATR, which activates checkpoint signalling upon genotoxic stresses, was downregulated in permanently SIV-infected T cells. ATR is also assumed to be required for appropriate DNA integration of HIV-1 viral DNA [77]. Interestingly, retroviral integration has recently been shown to induce apoptosis by the activation of the DNA-dependent protein kinase (DNA-PK). Downregulation of ATR followed by interruption of the integration process of viral DNA, therefore, might inhibit provirus formation and counteract programmed cell death in chronically SIV-infected T cells.

The results suggest that E2F1 activities have been repressed and consequently might contribute to cell survival by preventing viral integration, virus-induced apoptosis and growth arrest via some of its target genes. Furthermore, the elongation of the G1 phase to increase transcription from the retroviral LTR, a property suggested for activated p53 and E2F in acute infection, should not occur in chronically infected cells because of the assumed activity pattern [68]. For E2F4, the number of downregulated target genes was 15 in contrast to only 7 upregulated target genes (Additional file 3: Figure S4), which indicates a more inhibitory role of E2F4 in this context.

C-Myc downregulates or represses various cell adhesion genes (αLβ2, α3β1 integrins), differentiation specific genes, differentiation markers, cell cycle/growth arrest and DNA damage genes (GADDs, p27^KIPI^). In most cases, an INR sequence (initiator negative regulation) is present in the promoter of these genes. In contrast, genes upregulated by Myc are integral to DNA synthesis and cell cycle regulation and have canonical E-box elements and lack INR sites. Upregulation of these genes is accompanied by cellular transformation. The presence or absence of INR elements in myc-targeted genes suggests that Myc plays an eminent role in the transition from proliferating undifferentiated cells to quiescent fully differentiated cells [78]. C-myc activity was affected in chronically SIV-infected T cells as predicted by the *ICF* and might promote the survival of these cells.

Finally, EGR1 (early growth response protein 1) activity was also predicted by the *ICF* to be affected (Additional file 2: Figure S3 and Additional file 3: Figure S4). EGR1 is a transcriptional regulator functioning in differentiation and mitosis. HIV-Tat protein transactivates the EGR1 promoter and this interaction has been associated with neuronal deficiency caused by HIV-1. Furthermore, EGR1 expression is downregulated in the SIV infection of the hippocampus [79, 80].

### Other affected pathways and comparisons to known host factors in HIV replication

#### Deregulation of chromatin formation and epigenetic alterations

In order to obtain information on the activation/repression of expression by chromatin formation and epigenetic mechanisms, the expression patterns for factors involved in such processes were investigated. Interestingly, the expression of H2A genes (HIST3H2A, HIST2H2AA3), members of the histone family that wrap and compact DNA into chromatin, was upregulated in the C8166-P cell line (Figure 5A). Moreover, PLU-1 expression (putative chromatin-binding motif, also known as JARID1B), was upregulated in chronically infected cells (Figure 5A). PLU-1 represents a H3K4 histone demethylase that is involved in transcriptional regulation. SATB1 (special AT-rich binding protein 1) is a protein binding to DNA associated to the nuclear scaffold/matrix. SATB1 RNA abundance was lower in chronically SIV-infected T cells (data not shown). SATB1 serves as a docking site for several chromatin-remodelling enzymes (e.g. PML at the MHC-I locus) and recruits co-repressors or co-activators directly to promoters and enhancers. Similarly, ATF-2, which represents a transcription factor and probably exerts its effects on chromatin components, was affected (Additional file 8: Table S3). Taken together, chromatin formation and epigenetic regulation seem to be affected during the chronic infection of T cells, possibly leading to distinct alterations in gene expression.

#### Other affected sub-networks and caveats

In addition to the sub-networks discussed above, several other pathways or sub-networks were deregulated upon chronic SIV infection (Additional file 5: Figure S2). However, their involvement in SIV infection cannot be discussed in such detail as that above. In particular, we noted that parts of the T-cell receptor signalling network (CD3Z, the gene for the ζ subunit of the T-cell receptor) and parts of metabolic network, e.g. glycosylation pathways, were deregulated (Table 2). Interestingly, similar to CD3Z in an HIV-resistant T cell clone [81], CD3Z expression was upregulated in chronically SIV-infected T cells in our study. Finally, SMAD1 was identified as a top downregulated hub in C8166-P cells (Additional file 5: Figure S2, Figure 5B, Additional file 7: Table S2). SMADs transduce and modulate signals in multiple pathways (e.g. TGF-β, BMP) and influence cell growth, apoptosis, morphogenesis, development and immune response.

To some extent, differences in expression of single genes might arise from cell variability, since neither the chronically SIV producing cells were clonal, nor were the cells from the acute infection assay uniformly infected. Unfortunately, this kind of complexity is a general problem in the majority of whole transcriptome analyses except for the most-updated single cell RNA-Seq analyses. An alternative approach, isolation of newly infected cells is difficult to achieve, stresses the cells and gives rise to another unpredictable variable. Therefore, the current approach represents a compromise on technical feasibility and informative value. However, we think that the unspecific noise did not influence results achieved for the main pathways identified in our analysis. The chronically infected cells went through a selection process and surviving, SIV-releasing cells were outgrown. The heterogeneity is suspected to be quite low, since in the long term, the culture is dominated by cells with growth advantage.

#### Comparison to gene expression profiles from chronic and acute HIV-1 infection of human T cell lines

As expected, we found that several signal transduction pathways and functional gene clusters involved in acute and chronic infection had been identified in previous studies of HIV infection of T cell lines, such as apoptosis control, cell cycle control or chromatin modulation. Most prominently, the cell survival was accompanied by the loss of CD4 expression in many cases. Olivares et al. generated gene expression profiles of chronically HIV-infected H9 cells using cDNA microarrays [82]. When we compared these profiles to that of chronically SIV-infected C8166, 21 genes overlapped, a majority being involved to cell cycle control (7) and apoptosis (4) (data not shown). More than 50% of the gene overlaps were regulated in the same direction. Obviously, similar mechanisms seem to accompany the chronic infection with HIV-1 and SIV of human T cell lines. The affected members of the respective pathways were often not identical in the different, infected cell lines. For example, CFLAR (CASP8 and FADD-like apoptosis regulator, FLIP), CTCF (a chromatin modulator) and RBM34 (KIAA0117) previously identified in chronic HIV-1 infection of T cell lines H9 (CFLAR) and SupT1 (CTCF, RBM34) were not affected in chronic SIV infection of C8166 [81, 82]. This suggests that interference with a pathway is more important than regulation of a specific gene member. Finally, specific genes and gene families reported to be deregulated in the acute HIV infection of CD4^+^ T CEM cell line, such as certain genes in cholesterol biosynthesis, were not influenced in lytically or chronically SIV_mac_251-infected C8166 T cells [83, 84].

#### Deregulation of HIV-dependency factors (HDFs)

Host factors required for HIV replication have been identified recently by functional genomic screens [8, 85–87]. Unfortunately, little overlap is seen between the results of these studies; this is clearly attributable to differences in experimental design and scope [88]. In their RNAi screen, Yeung et al. have identified 252 human genes whose chronic knockdown in Jurkat cells apparently interrupts productive HIV-1 infection [87]. Interestingly, their results are mostly distinct from the HeLa and 293 T candidates described by Brass *et al.* [8], Koenig *et al.* [85], and Zhou *et al.* [86], possibly because a T cell line better reflects the physiological condition of an HIV infection than HeLa or 293 T cells.

**Figure 8:**
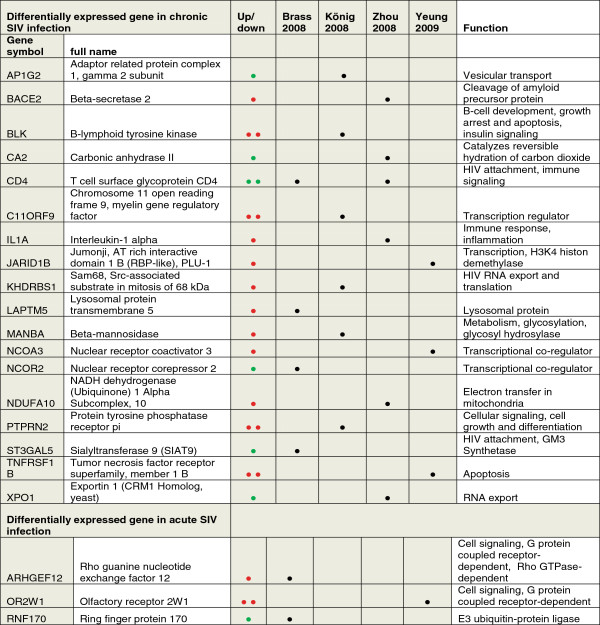
**Occurrence of differentially expressed genes from SIV-infected T-cells in RNAi screens for host factors required for HIV replication.** Differentially expressed (Diff) genes of acute and chronic SIV infection of T cells were identified in four published RNAi screens for HIV dependency factors. Diff presence is indicated by a filled circle. Additional symbols as in Figure 5.

Notwithstanding the discrepancies, these studies have prompted us to investigate whether any of these HDFs was differentially expressed in the chronically or acutely SIV-infected C8166. Out of the HDFs ranging in number from 233–295 detected in four investigations, 18 HDFs were differentially expressed in the permanently SIV-releasing cell line compared with the control (Figure 8). Three genes were found in the HDFs listed in Yeung et al., 4 in Brass et al., 6 in Koenig and colleagues and 6 in Zhou et al. Not surprisingly, only one HDF (CD4) was identified in two RNAi screens, and none was present in all four screens. In addition, only 3 genes were differentially regulated compared with the acutely infected cell.

The HDFs found in the chronically SIV-infected T cells function in HIV docking to the plasma membrane (CD4, SIAT9), cell cycle control (BLK, PTPRN2), apoptosis (BLK, TNFRSF1B), HIV RNA export (XPO1, SAM68) and immune signalling (CD4, IL1A). Four of them are transcriptional regulators (MRF, NCOR2, NCOA3, PLU-1). AP1G2, an adaptin, is part of the adaptor complex involved in clathrin-coated vesicle transport, whereas LAPTM5 and NDUFA10 reside in specific compartments (lysosomes, mitochondria).

Noteworthy, the caveolin contribution to HIV infection has also been observed in two functional genomic screens by Brass et al. and Zhou et al. The authors recently identified Cav-2, a Cav-1 relative that needs Cav-1 for transport and function, as one of hundreds of HDFs that are likely to play a relevant role in HIV pathogenesis in acute infection of HeLa-derived cell lines [8, 86, 89]. However, Cav-2 is not found in RNAi screens of infected 293 T or human lymphocytes.

Furthermore, 21 Diff genes of chronically SIV-infected T cells were identified in the VirusMINT database, a compilation of virus-interacting human proteins published in 2009 (data not shown) [69]. Among the subgroup of proteins interacting with HIV, we found Cav-1, CD4, CD9 and the complement receptor CR2, two histone genes, eight HLA genes (all major histocompatibility complex, class II), two RNA export factors (SAM68, XPO1), and the HIV Rev-binding protein HRB. Moreover, PLAU (plasminogen activator, urokinase type), PDIA5 (a protein disulfide isomerase) and ACTA2 (an α-actin) were also found.

Nucleoporins are part of the nuclear pore complex, which regulates the nucleo- cytoplasmic transport of molecules. Several nucleoporins (Nup 107, 133, 153,155, 160, 185) have been identified as HDFs (listed in [8]) and some of them are required for the uncoating and inward trafficking of the pre-integration complex. Notably, Nup205, a nucleoporin required for the long-term maintenance of the nuclear pore complex, was downregulated in chronically SIV-infected T cells (Figure 5A). NUP205 has been detected in DNA microarray analyses of chronically HIV-1 infected H9 T cell line [82] and has been noted as an HIV interaction partner in a proteomic screen involving affinity purification and mass spectrometry [90], but has not been identified in the 4 HDF screens through RNAi mentioned above. Apparently, NUP205 escaped the RNAi approaches; this might be because these screens covered only gene products that are not essential for cell survival. Thus, Nup205 might represent a novel factor, the downregulation of which is involved especially in establishing cellular resistance to SIV-replication in T cells.

## Conclusion

A conventional pathway analysis method and a new network-and-knowledge-based strategy were used in gene profiling experiments to identify potential key genes and sub-networks associated with cell survival in a chronic SIV-infection. Our results demonstrate that several complex sub-networks together, rather than a single pathway, play a role in the survival of the chronically SIV-infected human CD4+ T cell line. We have not only confirmed certain previously reported pathways, but also revealed several new sub-networks that might jointly contribute to the persistence mechanism of SIV infection of T cells.

In particular, by tracing the interaction networks, putative ‘hidden’ key regulators were identified in chronically infected T cells; such regulators were not significantly affected at the mRNA levels. We also identified differentially-expressed top hubs, e.g. caveolin-1, through the network analysis. Further experimental studies confirmed caveolin-1 upregulation and CD4 downregulation in the survival network of chronically SIV-infected human CD4 T cells. At a network level, the analyses revealed that chronically SIV-infected T cells releasing infectious virus particles counteracted various cellular processes that have been described to participate in the early phase of viral replication and cell death in acute infections. Re-infection of the chronically infected cell is probably prevented by reduced CD4 receptor availability, interference with alternate routes of viral entry, nuclear transport, integration of proviral DNA, RNA export and cell survival afforded by reprograming of apoptosis and cell growth. Late processes leading to the generation of new virus do not seem to be influenced to such an extent. This suggests that the high virus productivity of the chronically infected T cell line might be attributed to proviral transcription rather than to ongoing viral replication. The investigation raises several hypotheses and possible sub-networks/pathways that might contribute to cell survival of SIV infection but these await further experimental confirmation. With the help of the results from this study, single or combined gene expression/depletion and other functional experiments can now be designed to investigate further their impact on infection by SIV and HIV.

## Materials and methods

### Cell culture and virus assays

C8166 cells were grown in RPMI 1640 medium supplemented by 10% fetal bovine serum and antibiotics (R-10 medium). C8166 permanently SIVmac251/32H releasing cell line was kindly provided by Christiane Stahl-Hennig who also established this cell line, which produces a high titre of infectious SIV without any evidence of lytic destruction [91]. The cell line is not clonal, but heterogeneity is expected to be low, since in the longterm, the culture is dominated by cells exhibiting a growth advantage. For virus titration, 30,000 C8166 cells were seeded into each well of a 96-well plate in 50 μl medium. A tenfold serially diluted virus stock (100 μl) was added to each well in 8 replicates. After 4 days, 50 μl fresh medium was added. After 7 days, cells producing virus proteins were stained by an indirect immunoperoxidase assay [92, 93]. The number of infectious viruses (TCID50) was scored by using the Reed and Muench method [94].

For quantification of viral RNA copies from the cell supernatant, RNA was extracted following the MagAttract Viral RNA M48 Kit virus mini protocol (Qiagen, Germany). RT-PCR was carried out as described by using the ABI 7500 Real Time PCR System [95].

Quantification of the proviral load was carried out by using the same primers and PCR conditions as for RNA quantification, except that the RNA was substituted by 50 and 100 ng of genomic DNA, isolated at four time points over a period of five months, and the RNA standard was replaced by a DNA-based standard. For the determination of proviral copy number, we assumed that the amount of DNA per cell equalled to 6.6 pg.

To estimate the number of SIV-particles released per cell per 24 h, the C8166 cells were washed 5 times and finally cultured for 24 h at a density of 10^6^ cells per ml R-10 medium. RNA copy numbers were determined from the wash solutions and after 24 h. For a conservative estimate, the RNA copy numbers detected in the final wash solution were subtracted from the RNA copy numbers obtained after the 24 h culture. Finally, the RNA copy numbers were divided by two to calculate number of viral particles. The results represent the mean from three independent experiments.

Halo-FISH was performed as described in Goetze *et al*. by using the SIV GagEnv fragment of plasmid pGX10-SIV-GE [96].

### Infection and RNA isolation

For each microarray experiment, 3×10^7^ cells were harvested by centrifugation (10 min, 600×g). To minimize potential effects related to change of medium, un-infected and permanently SIV-producing C8166 were re-suspended using 20 ml old and 10 ml fresh R-10 medium, and incubated for 24 h at 37°C in a 5% CO2 humidified chamber. For the acute infection, 3×10^7^ un-infected C8166 cells were harvested and re-suspended in 20 ml stock solution of SIVmac251 (multiplicity of infection: 0.2) grown on C8166 cells in R-10 medium and 10 ml fresh R-10 medium. Cells were incubated for 24 h at 37°C in a 5% CO2 humidified chamber. After 24 h, cells were harvested and processed for immediate RNA isolation. The number of viral particles present in the virus stock solution was determined by real-time PCR. Comparing the results of the real-time PCR with those obtained by virus titration revealed a ratio of non-infectious: infectious viral particles of about 4000:1. Each cell was thus theoretically in contact with about 800 virus particles which reflect the number of virus particles present in the supernatant (i.e. “old” medium) of the permanently SIV-producing cell line.

Total RNA was isolated by using PeqGold TriFast FL RNA isolation kit (PeqLab) and quality was checked by formaldehyde gel electrophoresis. Prior to microarray analysis, a second RNA quality check was performed by an Agilent Bioanalyzer.

### Microarray analysis

The RNA was labelled and hybridized to Affymetrix microarrays. All the microarrays included in this work met broadly accepted quality control standards, including present call percentage (40-49%, an ideal spread <10%), GAPDH 3’/5’ or 3’/M ratios (0.77-1.0, ideally to be <4) and normalized unscaled standard errors (NUSE) median (all <1.02, ideally to be < 1.1). According to the Affymetrix recommendation, background levels should be less than 100 (our arrays spread from 63 to 89). Furthermore, in this study, BioB controls received present calls for all the 6 samples, indicating good detection levels of even low abundance mRNA and low noise. The intensity levels for BioB, BioC, BioD and Bio-cre showed increasing expression in all the arrays as expected, indicating a good hybridization process.

The expression ratios for each mRNA transcript on Affymetrix HG U133A genechips (25 mer oligonucleotide probe sets) between samples and controls were determined by the software Microarray Suite 5.0 (Robert Geffers, HZI). First, all the transcriptional probe sets (322) were excluded corresponding to more than one gene according to the annotation of Affymetrix chip probe sets from Affymetrix website. In this work, we performed two independent experiments. To assure safer and more reliable results, we only filtered for the transcriptional probe sets with the same detection call (according to one-sided Wilcoxon Signed-Rank test, p < =0.05 is present, p > =0.065 is absent and between them is marginal) in the two repetitions. The detection call was also assigned as being marginal, if the detection call of one probe was absent or present in one experiment but was marginal in the other experiment. Subsequently, both absent genes (AA) in the given sample and control were excluded. If a transcript was detected with more than one probe, the expression change of the probe with highest response was regarded as the expression change of the given gene. Changes in expression levels equal to or greater than two-fold (corresponding to 1 or -1 after log2 transformation) after SIV infection were regarded as significantly differentially expressed. Otherwise, they were considered as NDiff genes.

### Integrated molecular networks of human cells

The conventional methods are restricted to identifying candidate genes that are differentially expressed under conditional or pathogenic changes. However, many key regulators exert their effects mainly through post-transcriptional and/or post-translational modifications. Thus, they are not recorded by standard DNA-microarray analysis. To address this issue, we developed a new approach, termed *Inter-Chain-Finder (ICF)*, that can be used (1) to find potential interactive molecular chains (IMCs) affecting the activities of NDiff regulators, (2) to identify the ‘hidden’ key regulators and (3) to uncover the integrated responsive network. The *ICF* is based on an integrated large-scale molecular network and the tracing of responsive pathways/network and IMCs.

To establish an integrated molecular network for human cells, we here uniformed all the related interaction data from the databases such as KEGG (http://www.genome.jp/kegg/), BIND (http://www.bindingdb.org) and HPRD (http://www.hprd.org). For the BIND database, all the interactions between human and other organisms were removed. We also excluded the interactions between genes (proteins) and small molecules (e.g. metabolites). Although these interactions might have been useful regarding feedback regulation, they were not included, as this work was mainly based on microarray data. Furthermore, the interactions between genes (proteins) and protein complexes were not taken into account. In the BIND database many interactions were removed because genes were deleted or discontinued according to the NCBI GenBank database. Some interactions with genes (proteins) also had gene information ID (GI) but without matched Entrez GeneID. These interactions were also excluded. Some proteins were not assigned GeneID by BIND, although they had ID according to GenBank. We systematically checked them and assigned them corresponding GeneIDs. These interactions were divided into two categories: protein-protein interactions (PPIs) and protein-DNA interactions (PDs), namely potential gene regulatory interactions.

From the KEGG database, 39113 interactions were obtained. Among them, 23291 were enzyme-enzyme relationships (EERs), meaning that two enzymes catalyze successive reactions. Theses interactions form the metabolic network in which nodes represent enzymes. The representation of a metabolic network is similar to the reaction network representation [97]. Together, 15822 interactions were PPIs or PDs. Since the KEGG data are based on pathway information, all the interactions are assigned a pathway index, whereas the interactions from BIND and HPRD do not have this attribute. These indexes have been used for affected pathways/sub-networks calculation and identification. In addition, all the PPIs from KEGG have more detailed information about the directed relationships such as activation, inhibition, phosphorylation or ubiquitination etc. and PDs have specific relationships, e.g. expression or repression. IMCs can be traced with these directed and undirected PPIs. The entire regulatory network (12146 interactions) is directed from one regulator to one targeted gene.

Subsequently, the interactions from BIND, HPRD and KEGG were compared to exclude repetitions. Because EERs uniquely exist in KEGG, they were not included in the comparison. Although a large number of additional PPIs from BIND and HPRD were added, all these extra PPIs were undirected. Most of the PPIs from KEGG had the direction, except for those with association or dissociation relationships. To obtain more complete information on interactions, peer-reviewed literature was mined and some additional interactions, especially for the mainly studied gene Cav-1, were added manually in this study. Finally, 86516 interactions with 11976 genes were obtained that included PPI, PD and EER.

For further analysis, the integrated network consisting of PD, PPI and EER interactions was correspondingly divided into three parts: transcription regulatory network, signal transduction network (including protein-protein interactions) and metabolic network. Since the three parts interact with each other, the method can comprehensively analyse the mutual effects among them, e.g. the way that the PD network controls the PPI or EER network or the manner in which the PPI network controls the PD network.

### Present and affected signal transduction sub-network

If both genes of a given signal interaction pair are present in the sample according to detection call of Microarray Suite 5.0, this interaction is present. Because it is difficult to determine which expression level can function in signal transduction, we also consider the signal interactions with marginally expressed genes as being ‘present’. If at least one gene of a present signal interaction is differentially expressed, we term it as a significantly affected interaction. The significantly affected interactions constitute the significantly affected signal transduction network. The definitions concerning the signal network in the sample are also applied to the control.

### Significantly affected regulatory sub-network

If both the regulator and the targeted gene are significantly differentially expressed, the PD is apparently significantly affected. If the targeted gene is differentially expressed but the regulator is not, the interaction is also potentially significantly affected since the activities of transcriptional factors are often modulated via posttranscriptional or posttranslational modification or ligand binding. Although the signal transduction network is far from being complete, we assume, in this work, that a regulator is not significantly affected if no corresponding significantly affected signal transduction chain can be found for the regulator. Since mRNA expression of some regulators was either present/marginal in the infected but not in the control samples, or *vice versa*, the significantly affected signal transduction chains were traced separately in control and treated samples.

### Significantly affected metabolic sub-network

If both genes in one EER are differentially expressed, this interaction is termed as a significantly affected metabolic interaction. A significantly affected metabolic interaction is defined in the conservative way stated above, which simplifies the complex theory of metabolic control. The significantly affected metabolic network is made up of significantly affected EERs.

### Principle of the tracing algorithm for possibly affected interactive molecular chains

The tracing algorithm searches for the upstream effectors that might modulate the activity of the given regulator and minimizes uncertainties arising from undirected protein-protein interactions (e.g., from BIND).

In the first step, all PPIs are identified that are linked to a given non-differentially-expressed regulator. These PPIs represent the first level (Figure 2B) of possible, significantly affected IMCs, which might modulate the activity of the given regulator. Directed PPIs (e.g. with activation or inhibition relationship) with significantly differentially expressed gene(s) are considered to most likely affect the activity of the given regulator. For undirected PPIs (e.g. from BIND or HPRD), the differentially-expressed gene products can bind to the given regulator and hence also possibly affect the regulatory activity of the given regulator but with some uncertainties regarding the direction. In the following paragraph, an approach is proposed to compensate for these uncertainties.

This tracing algorithm is aimed at finding the most direct (shortest), significantly affected IMCs that might modulate the activity of the given regulator. Two types of cases exist: IMCs with or without undirected PPIs. If all the PPIs in the first level linking the non-differentially-expressed regulator and differentially-expressed genes are directed, the search is not switched to the next level and the resulting IMCs are composed of only those directed PPIs with the chain length of one. On the other hand, if some PPIs that link the non-differentially-expressed regulator and differentially-expressed genes are undirected, we extend the interaction chains for directed/undirected PPIs that link the non-differentially-expressed regulator and non-differentially-expressed genes (“ND-PPIs”) from the first level by searching for directed PPIs in the second level (Figure 2B) which connect differentially-expressed genes and non-differentially-expressed genes from ND-PPIs of the first level. For convenience, we call PPIs linking differentially-expressed genes with non-differentially-expressed genes as “Diff-PPIs”. From the second level or higher levels, we only search for directed PPIs. Thereafter, we perform the core steps: if any directed Diff-PPI from the second level is linked to a ND-PPI, the search is not switched to the next level. The search is continued for the given ND-PPI to identify all the other directed Diff-PPIs in the second level, which link to that ND-PPI. This iteration process is performed for each identified ND-PPI from the first level. The resulting IMCs have the chain length of two.

If only directed ND-PPIs but no directed Diff-PPIs from the second level are found for any ND-PPI from the first level, we switch to the next higher level until any directed Diff-PPI linking to any ND-PPI is found in the current level, where we would repeat the core steps as described above. The chain length of the resulting directed IMCs is in the order of the current level in which we have found the Diff-PPIs (see Figure 2B and as exampled in Additional file 2: Figure S3). All the potential, significantly affected IMCs for the given regulator can be found by the above algorithm.

### Statistical analysis

#### Assessment of the statistical significance of affected regulators and sub-networks

We further calculated whether the number of PDs affected by a given regulator (or the number of affected PPIs and EERs) was obtained by chance or not, as only being affected by the IMCs is not sufficient to judge the significance of the given regulator. The binomial distribution was used to calculate the P-value, e.g., the chance that m_ex_ PDs were found to be regulated by the given regulator in the affected network extracted from all the available regulatory network was calculated by the formula:
1

Where: n, number of PDs in all the available regulatory network; m, number of all the known PDs regulated by the given regulator; n_ex_, number of PDs existing in the affected regulatory network; m_ex_, number of PDs in this extracted network regulated by the given regulator.

If the significance for the given regulator is high, the number of PD found to be regulated by this regulator is not obtained by chance.

Analogously, we assessed the number of affected PPIs or EERs that belong to given signalling transduction or metabolic sub-networks expected under the null hypothesis. The significance that m_ex_ EERs were found to belong to the given sub-network in the affected metabolic network could also be calculated by Equation 1. Similarly, the significance for the affected signaling transduction sub-networks according to KEGG was obtained by Equation 1. The sub-networks in the highest ranking positions ordered by significance values were the most possibly affected sub-networks.

#### Calculation of the enrichment of genes in human GO processes

A module of the *ICF* that is not included in the GO-viewer software calculates the enrichment of genes in the human GO processes and, thus, investigates the detailed functions for the protein-protein network and regulatory network. For each human GO biological process, the probability that the observed number of genes could be found by chance was determined by calculating the cumulative binomial distribution probability. In this work, the calculation of probability is different from that in most of other GO terms searching programs, e.g. GeneMerge [98]. We calculate the probability of processes in the given degrees of the GO hierarchical tree instead of all the GO terms in the whole tree. Suppose that there are n annotated genes in the given GO process of the hierarchical tree (in this work, the degree from four to nine is considered). Among them, m genes are assigned to a given GO process term. n_ex_ genes exist in the test sample. In this test sample, m_ex_ genes are known to be involved in the given GO process. The significance can also be calculated by Equation 1.

### Lysis of cells, SDS-PAGE and Western blot analysis

Cells were treated with lysis buffer (10 mM Tris pH7.5, 50 mM NaCl, 1% Triton X100, 60 mM octylglucoside (Roche), 1 mM aprotinin, 1 mM leupeptin, 1 mM PMSF) at 4°C for 30 min followed by centrifugation in an Eppendorf centrifuge at 15000 rpm. Cleared supernatants were processed further or stored at -20°C. SDS-PAGE and Western Blot analysis for caveolin-1 detection were performed as described previously [52].

### Real-time RT-PCR

RT-PCR was performed from equal amounts of total RNA (640 ng) by using LighCycler™ technology and a RT-PCR amplification kit (Roche Diagnostics). Caveolin-1 specific signals were obtained by intron-spanning RT-PCR (forward primer hCav1_54 GAA AGA GAG AAT GGC GAA GTA; reverse primer hCAV1_371 CGG GAA CAG GGC AAC ATC TAC, PCR product mRNA calculated length 318 bp). Values were normalized by using probes for RT PCR of the house keeping gene for ribosomal protein S9 (RPS9) kindly provided by Dr. Wiebke Hansen (forward primer 5′- CGC AGG CGC AGA CGG TGG AAG C, reverse primer CGA AGG GTC TCC GCG GGG TCA CAT). For confirmation of product identity, the amplification products were separated by 2% agarose gel electrophoresis.

Expression levels of other transcripts were determined using the ABI Prism 7500 Real Time PCR System. 1 μg of total RNA was additionally purified with gDNA elimination spin columns (Qiagen, Germany) and reverse transcribed by using the QuantiTect reverse transcription kit (Qiagen, Germany). Four serial dilutions of the cDNA were amplified in duplicate by using commercially available primers (Gene globe, Qiagen, Germany) and the ImmoMix polymerase kit (Bioline, Germany) supplemented with Rox and Sybr Green. As standard, serially diluted cDNA from HumanXpress Ref universal reference total RNA (Superarray Bioscience Corporation) was employed. The reciprocal dilution factor of the standard was set arbitrarily as “copy number”. As further controls, RPS9 expression was measured in each sample. The relative “copy numbers” were compared with those of uninfected C8166 cells and represent the mean of all measurements. The results represent the mean of two independent experiments.

### Flow cytometry

Surface expression of CD4 was determined by flow cytometry. Uninfected and chronically infected C-8166 cells were incubated for 30 min at 4°C in the dark with an antibody directed against CD4 coupled to PE (clone M-T477, BD Biosciences) at pretitrated concentrations in phosphate-buffered saline (PBS) with 1% bovine serum albumin and 5% heat-inactivated normal mouse serum to block unspecific binding. Subsequently, cells were washed, pelleted by centrifugation and resuspended in 3% formaldehyde for fixation. Samples were measured on an LSRII flow cytometer (BD Biosciences) and data were analysed by using FlowJo software (Tree Star, Ashland, OR).

## Availability of supporting data and programs

The complete microarray dataset is available in the Gene Expression Omnibus (GSE51594). The integrated database and the *ICF* program are available in the webpage http://www.uni.lu/lcsb/publications/integrative_network_analyzer.

## Electronic supplementary material

Additional file 1: Figure S1: Virus produced by C8166-P cells (chronically SIV-infected cells) replicates similarly compared to highly pathogenic SIVmac strains and causes a lytic infection. Description: In vitro viral replication pattern of different SIV virus strains: **(a)** viral RNA copies in cell culture supernatant of in vitro infected C8166 cells, **(b)** viral RNA copies in cell culture supernatant of in vitro infected rhesus monkey peripheral blood mononuclear cells (PBMCs). Before infection, C8166 cells and rhesus PBMCs were washed. 2x106 cells were re-suspended in 2 ml cell culture medium containing 50,000 TCID50 of SIVmac251/32H (supernatant of C8166-P), or 60,000 TCID50 SIVmac239 grown on C8166 cells or 30,000 TCID50 SIVmac251 grown on rhesus monkey lymphocytes. After an incubation step for 2 h at room temperature, cells were washed twice and cultured in 1 ml RPMI 1640 medium (PAN Biotech, Aidenbach, Germany) supplemented with 10% fetal calf serum (PAN Biotech, Aidenbach, Germany), 100 U/ml penicillin (PAN Biotech, Aidenbach, Germany) and 100 μg/ml streptomycin (PAN Biotech, Aidenbach, Germany; complete RPMI 1640). Before infection, frozen rhesus monkey PBMCs were thawed and stimulated with 2.5 μg ConA/ml overnight. Infection with different SIV strains was performed as described above. After infection, monkey PBMCs were cultured in RPMI 1640 complete medium supplemented with 100 U/ml recombinant human IL-2 (PeproTech, Hamburg, Germany). Viral RNA copies in cell culture supernatant were determined at day 3 and 6 post infection as described in Materials and Methods. Syncytia as evidence of lytic infection are observed after infection of C8166 cells (**c**) and PBMCs (**d**) with supernatant of C8166-P. (PPTX 241 KB)

Additional file 2: Figure S3: Significantly affected interactive molecular chains (IMCs) from chronically SIV-infected samples, which might modulate the activity of the ‘hidden’ key regulators. Different ‘hidden’ key regulators exhibiting no change in their RNA level are inferred by tracing the upstream interactive molecular network. Regulators are represented by diamonds. The black delta represents activation; the black circle represents inhibition; a simple line without a symbol represents an undirected relationship PPI of the two proteins. Red indicates significantly increased, green, significantly decreased. (PDF 60 KB)

Additional file 3: Figure S4: Parts of putative, non-differentially expressed key regulators and the affected transcription regulatory network identified by the *ICF*. The figure shows genes that are regulated at the RNA level in the chronically SIV- infected T cell line by non-differentially expressed (NDiff), putative key regulators. The direction of the arrow is from the regulator to the target gene. The target genes of E2F1 are indicated by hexagons. For a detailed discussion of the effects of P53, Myc, E2F and p53 on target genes and the cell, see text. The activity of transcription factor CREB is accompanied by the upregulation of members of the MHC class II family (HLA-D) and the downregulation of cyclin A 2 (CCNA2), a protein that activates certain kinases to promote cell cycle transitions. (PDF 30 KB)

Additional file 4: **Changes in gene expression in chronically and acutely SIV- infected C8166 T cells.** The file lists up- and downregulated genes in chronically and acutely SIV-infected C8166 cells and the degree of regulation (changes in gene expression, log2 transformed from fold changes, versus mock-infected control cells). (DOCX 28 KB)

Additional file 5: Figure S2: Protein-protein network of CD4+ T cells significantly perturbed only in the chronically SIV-infected cells identified by the *ICF*. Affected sub-networks in chronically infected C8166 cells are shown. Red colour, significantly increased; green, significantly decreased; white, non-differentially expressed (NDiff). (PDF 539 KB)

Additional file 6: Table S1: Top upregulated hubs in the chronic SIV infection of C8166 T cells predicted by the *ICF.* (XLSX 12 KB)

Additional file 7: Table S2: Top downregulated hubs in the chronic SIV infection of C8166 T cells predicted by the *ICF.* (XLSX 12 KB)

Additional file 8: Table S3: Top ‘hidden’ key regulators in chronically SIV-infected C8166 T cells predicted by the *ICF.* (XLSX 12 KB)

## Abbreviations

Cav-1: Caveolin-1
CD3Z: Zeta-subunit T-cell receptor
CD4: Cluster of differentiation 4, T-cell surface glycoprotein
CD44: Cluster of differentiation 44, hyaluronic acid receptor
FUT7: Fucosyltransferase VII
C-Myc: v-myc myelocytomatosis viral oncogene homolog (avian)
Diff: Significantly differentially expressed
EER: Enzyme-enzyme relationship
E2F1: E2F transcription factor 1
E2F4: E2F transcription factor 4
HDF: HIV-dependency factor
HIV: Human immunodeficiency virus
ICF: Inter-Chain-Finder
IL2RA: Alpha-subunit interleukin-2 receptor
IMC: Interactive molecular chain
MAPK: Mitogen-activated protein kinase
NDiff: Non-differentially expressed
PD: Transcription regulatory interaction or protein-DNA interaction
PPI: Protein-protein interaction
SIV: Simian immunodeficiency virus
TNF-α: Tumor necrosis factor α
TP53: Tumor protein p53.

## References

[1] Rotger M, Dalmau J, Rauch A, McLaren P, Bosinger SE, Martinez R, Sandler NG, Roque A, Liebner J, Battegay M, Bernasconi E, Descombes P, Erkizia I, Fellay J, Hirschel B, Miró JM, Palou E, Hoffmann M, Massanella M, Blanco J, Woods M, Günthard HF, De Bakker P, Douek DC, Silvestri G, Martinez-Picado J, Telenti A (2011). Comparative transcriptomics of extreme phenotypes of human HIV-1 infection and SIV infection in sooty mangabey and rhesus macaque. J Clin Investig.

[2] Kaslow RA, Dorak T, Tang JJ (2005). Influence of host genetic variation on susceptibility to HIV type 1 infection. J Infect Dis.

[3] Donfack J, Buchinsky FJ, Post JC, Ehrlich GD (2006). Human susceptibility to viral infection: the search for HIV-protective alleles among Africans by means of genome-wide studies. AIDS Res Hum Retroviruses.

[4] Goff SP (2007). Host factors exploited by retroviruses. Nat Rev Microbiol.

[5] Lama J, Planelles V (2007). Host factors influencing susceptibility to HIV infection and AIDS progression. Retrovirology.

[6] Arganaraz ER, Schindler M, Kirchhoff F, Cortes MJ, Lama J (2003). Enhanced CD4 Down-modulation by Late Stage HIV-1 nef Alleles Is Associated with Increased Env Incorporation and Viral Replication. J Biol Chem.

[7] Lama J (2003). The physiological relevance of CD4 receptor down-modulation during HIV infection. Curr HIV Res.

[8] Brass A, Dykxhoorn DM, Benita Y, Yan N, Engelman A, Xavier RJ, Lieberman J, Elledge SJ (2008). Identification of host proteins required for HIV infection through a functional genomic screen. Science.

[9] George MD, Sankaran S, Reay E, Gelli AC, Dandekar S (2003). High-throughput gene expression profiling indicates dysregulation of intestinal cell cycle mediators and growth factors during primary simian immunodeficiency virus infection. Virology.

[10] Giri MS, Nebozhyn M, Showe L, Montaner LJ (2006). Microarray data on gene modulation by HIV-1 in immune cells: 2000–2006. J Leukoc Biol.

[11] Ndolo T, George M, Nguyen H, Dandekar S (2006). Expression of simian immunodeficiency virus Nef protein in CD4+ T cells leads to a molecular profile of viral persistence and immune evasion. Virology.

[12] Li Y, Chan EY, Katze MG (2007). Functional genomics analyses of differential macaque peripheral blood mononuclear cell infections by human immunodeficiency virus-1 and simian immunodeficiency virus. Virology.

[13] Mehla R, Ayyavoo V (2012). Gene array studies in HIV-1 infection. Curr HIV/AIDS Rep.

[14] Lederer S, Favre D, Walters KA, Proll S, Kanwar B, Kasakow Z, Baskin CR, Palermo R, McCune JM, Katze MG (2009). Transcriptional profiling in pathogenic and non-pathogenic SIV infections reveals significant distinctions in kinetics and tissue compartmentalization. PLoS Pathog.

[15] Bosinger SE, Li Q, Gordon SN, Klatt NR, Duan L, Xu L, Francella N, Sidahmed A, Smith AJ, Cramer EM, Zeng M, Masopust D, Carlis JV, Ran L, Vanderford TH, Paiardini M, Isett RB, Baldwin DA, Else JG, Staprans SI, Silvestri G, Haase AT, Kelvin DJ (2009). Global genomic analysis reveals rapid control of a robust innate response in SIV-infected sooty mangabeys. J Clin Investig.

[16] Rud EW, Cranage M, Yon J, Quirk J, Ogilvie L, Cook N, Webster S, Dennis M, Clarke BE (1994). Molecular and biological characterization of simian immunodeficiency virus macaque strain 32H proviral clones containing nef size variants. J Gen Virol.

[17] Jung A, Maier R, Vartanian JP, Bocharov G, Jung V, Fischer U, Meese E, Wain-Hobson S, Meyerhans A (2002). Multiply infected spleen cells in HIV patients. Nature.

[18] Weissman D, Rabin RL, Arthos J, Rubbert A, Dybul M, Swofford R, Venkatesan S, Farber JM, Fauci AS (1997). Macrophage-tropic HIV and SIV envelope proteins induce a signal through the CCR5 chemokine receptor. Nature.

[19] Calvano SE, Xiao W, Richards DR, Felciano RM, Baker HV, Cho RJ, Chen RO, Brownstein BH, Cobb JP, Tschoeke SK, Miller-Graziano C, Moldawer LL, Mindrinos MN, Davis RW, Tompkins RG, Lowry SF (2005). A network-based analysis of systemic inflammation in humans. Nature.

[20] Diez D, Goto S, Fahy J, Erle D, Woodruff P, Wheelock A, Wheelock C (2012). Network analysis identifies a putative role for the PPAR and type 1 interferon pathways in glucocorticoid actions in asthmatics. BMC Med Genomics.

[21] Ogami K, Yamaguchi R, Imoto S, Tamada Y, Araki H, Print C, Miyano S (2012). Computational gene network analysis reveals TNF-induced angiogenesis. BMC Syst Biol.

[22] Daraselia N, Wang Y, Budoff A, Lituev A, Potapova O, Vansant G, Monforte J, Mazo I, Ossovskaya VS (2012). Molecular signature and pathway analysis of human primary squamous and adenocarcinoma lung cancers. Am J Cancer Res.

[23] Dennis G, Sherman BT, Hosack DA, Yang J, Gao W, Lane HC, Lempicki RA (2003). DAVID: Database for Annotation, Visualization, and Integrated Discovery. Genome Biol.

[24] Wei BL, Arora VK, Foster JL, Sodora DL, Garcia JV (2003). In vivo analysis of Nef function. Curr HIV Res.

[25] Wildum S, Schindler M, Muench J, Kirchhoff F (2006). Contribution of Vpu, Env, and Nef to CD4 down-modulation and resistance of human immunodeficiency virus type 1-infected T cells to superinfection. J Virol.

[26] Levesque K, Finzi A, Binette J, Cohen E (2004). Role of CD4 receptor down-regulation during HIV-1 infection. Curr HIV Res.

[27] Rose JJ, Janvier K, Chandrasekhar S, Sekaly RP, Bonifacino JS, Venkatesan S (2005). CD4 down-regulation by HIV-1 and simian immunodeficiency virus (SIV) Nef proteins involves both internalization and intracellular retention mechanisms. J Biol Chem.

[28] Jabbar MA, Nayak DP (1990). Intracellular interaction of human immunodeficiency virus type 1 (ARV-2) envelope glycoprotein gp160 with CD4 blocks the movement and maturation of CD4 to the plasma membrane. J Virol.

[29] Benson RE, Sanfridson A, Ottinger JS, Doyle C, Cullen BR (1993). Downregulation of cell-surface CD4 expression by simian immunodeficiency virus Nef prevents viral super infection. J Exp Med.

[30] Grigorov B, Arcanger F, Roingeard P, Darlix JL, Muriaux D (2006). Assembly of Infectious HIV-1 in Human Epithelial and T-Lymphoblastic Cell Lines. J Mol Biol.

[31] Little SJ, Riggs NL, Chowers MY, Fitch NJS, Richman DD, Spina CA, Guatelli JC (1994). Cell surface CD4 downregulation and resistance to superinfection induced by a defective provirus of HIV-1. Virology.

[32] Gallay P (2004). Syndecans and HIV-1 pathogenesis. Microbes Infect.

[33] Roscic-Mrkic B, Fischer M, Leemann C, Manrique A, Gordon CJ, Moore JP, Proudfoot AEI, Trkola A (2003). RANTES (CCL5) uses the proteoglycan CD44 as an auxiliary receptor to mediate cellular activation signals and HIV-1 enhancement. Blood.

[34] Saphire ACS, Bobardt MD, Zhang Z, David G, Gallay PA (2001). Syndecans serve as attachment receptors for human immunodeficiency virus type 1 on macrophages. J Virol.

[35] Wahl SM, Greenwell-Wild T, Peng G, Ma G, Orenstein JM, Vazquez N (2003). Viral and host cofactors facilitate HIV-1 replication in macrophages. J Leukoc Biol.

[36] Wahl SM, Greenwell-Wild T, Vazquez N (2006). HIV accomplices and adversaries in macrophage infection. J Leukoc Biol.

[37] Appelmelk BJ, Van Die I, Van Vliet SJ, Vandenbroucke-Grauls CMJE, Geijtenbeek TBH, Van Kooyk Y (2003). Cutting edge: Carbohydrate profiling identifies new pathogens that interact with dendritic cell-specific ICAM-3-grabbing nonintegrin on dendritic cells. J Immunol.

[38] Guo Y, Feinberg H, Conroy E, Mitchell DA, Alvarez R, Blixt O, Taylor ME, Weis WI, Drickamer K (2004). Structural basis for distinct ligand-binding and targeting properties of the receptors DC-SIGN and DC-SIGNR. Nat Struct Mol Biol.

[39] den Dunnen J, Gringhuis SI, Geijtenbeek TBH (2008). Innate signaling by the C-type lectin DC-SIGN dictates immune responses. Cancer Immunol Immunother.

[40] Hodges A, Sharrocks K, Edelmann M, Baban D, Moris A, Schwartz O, Drakesmith H, Davies K, Kessler B, McMichael A, Simmons A (2007). Activation of the lectin DC-SIGN induces an immature dendritic cell phenotype triggering Rho-GTPase activity required for HIV-1 replication. Nat Immunol.

[41] De Parseval A, Bobardt MD, Chatterji A, Chatterji U, Elder JH, David G, Zolla-Pazner S, Farzan M, Lee TH, Gallay PA (2005). A highly conserved arginine in gp120 governs HIV-1 binding to both syndecans and CCR5 via sulfated motifs. J Biol Chem.

[42] Rawat SS, Gallo SA, Eaton J, Martin TD, Ablan S, KewalRamani VN, Wang JM, Blumenthal R, Puri A (2004). Elevated expression of GM3 in receptor-bearing targets confers resistance to human immunodeficiency virus type 1 fusion. J Virol.

[43] Bobardt MD, Chatterji U, Selvarajah S, Van Der Schueren B, David G, Kahn B, Gallay PA (2007). Cell-free human immunodeficiency virus type 1 transcytosis through primary genital epithelial cells. J Virol.

[44] He F, Chen H, Probst-Kepper M, Geffers R, Eifes S, Del Sol A, Schughart K, Zeng AP, Balling R (2012). PLAU inferred from a correlation network is critical for suppressor function of regulatory T cells. Mol Syst Biol.

[45] Della Gatta G, Palomero T, Perez-Garcia A, Ambesi-Impiombato A, Bansal M, Carpenter ZW, De Keersmaecker K, Sole X, Xu L, Paietta E, Racevskis J, Wiernik PH, Rowe JM, Meijerink JP, Califano A, Ferrando AA (2012). Reverse engineering of TLX oncogenic transcriptional networks identifies RUNX1 as tumor suppressor in T-ALL. Nat Med.

[46] Wu C, Yosef N, Thalhamer T, Zhu C, Xiao S, Kishi Y, Regev A, Kuchroo VK (2013). Induction of pathogenic TH17 cells by inducible salt-sensing kinase SGK1. Nature.

[47] Bando SY, Alegro MC, Amaro E, Silva AV, Castro LH, Wen HT, Lima LA, Brentani H, Moreira-Filho CA (2011). Hippocampal CA3 transcriptome signature correlates with initial precipitating injury in refractory mesial temporal lobe epilepsy. PLoS One.

[48] Hatanaka M, Maeda T, Ikemoto T, Mori H, Seya T, Shimizu A (1998). Expression of caveolin-1 in human T cell leukemia cell lines. Biochem Biophys Res Commun.

[49] Fra AM, Williamson E, Simons K, Parton RG (1995). De novo formation of caveolae in lymphocytes by expression of VIP21- caveolin. Proc Natl Acad Sci U S A.

[50] Tomassian T, Humphries LA, Liu SD, Silva O, Brooks DG, Miceli MC (2011). Caveolin-1 orchestrates TCR synaptic polarity, signal specificity, and function in CD8 T cells. J Immunol.

[51] Liu P, Rudick M, Anderson RGW (2002). Multiple functions of caveolin-1. J Biol Chem.

[52] Yu Z, Beer C, Koester M, Wirth M (2006). Caveolin-1 interacts with the Gag precursor of murine leukaemia virus and modulates virus production. Virol J.

[53] Zou P, Wu F, Lu L, Huang JH, Chen YH (2009). The cytoplasmic domain of influenza M2 protein interacts with caveolin-1. Arch Biochem Biophys.

[54] Sun L, Hemgard GV, Susanto SA, Wirth M (2010). Caveolin-1 influences human influenza A virus (H1N1) multiplication in cell culture. Virol J.

[55] Ravid D, Leser GP, Lamb RA (2010). A role for caveolin 1 in assembly and budding of the paramyxovirus parainfluenza virus 5. J Virol.

[56] Nystrom FH, Chen H, Cong LN, Li Y, Quon MJ (1999). Caveolin-1 interacts with the insulin receptor and can differentially modulate insulin signaling in transfected Cos-7 cells and rat adipose cells. Mol Endocrinol.

[57] Simmons GE, Taylor HE, Hildreth JEK (2012). Caveolin-1 suppresses Human Immunodeficiency virus-1 replication by inhibiting acetylation of NF-κB. Virology.

[58] Llano M, Kelly T, Vanegas M, Peretz M, Peterson TE, Simari RD, Poeschla EM (2002). Blockade of human immunodeficiency virus type 1 expression by Caveolin-1. J Virol.

[59] Hovanessian AG, Briand JP, Said EA, Svab J, Ferris S, Dali H, Muller S, Desgranges C, Krust B (2004). The caveolin-1 binding domain of HIV-1 glycoprotein gp41 is an efficient B cell epitope vaccine candidate against virus infection. Immunity.

[60] Rey-Cuille MA, Svab J, Benferhat R, Krust B, Briand JP, Muller S, Hovanessian AG (2006). HIV-1 neutralizing antibodies elicited by the candidate CBD1 epitope vaccine react with the conserved caveolin-1 binding motif of viral glycoprotein gp41. J Pharm Pharmacol.

[61] Benferhat R, Sanchez-Martinez S, Nieva JL, Briand JP, Hovanessian AG (2008). The immunogenic CBD1 peptide corresponding to the caveolin-1 binding domain in HIV-1 envelope gp41 has the capacity to penetrate the cell membrane and bind caveolin-1. Mol Immunol.

[62] Huang JH, Lu L, Lu H, Chen X, Jiang S, Chen YH (2007). Identification of the HIV-1 gp41 core-binding motif in the scaffolding domain of caveolin-1. J Biol Chem.

[63] Wang XM, Nadeau PE, Lo YT, Mergia A (2010). Caveolin-1 modulates HIV-1 envelope-induced bystander apoptosis through gp41. J Virol.

[64] Lin S, Wang XM, Nadeau PE, Mergia A (2010). HIV infection upregulates caveolin 1 expression to restrict virus production. J Virol.

[65] Wang XM, Nadeau PE, Lin S, Abbott JR, Mergia A (2011). Caveolin 1 inhibits HIV replication by transcriptional repression mediated through NF-κB. J Virol.

[66] Lin S, Nadeau PE, Wang X, Mergia A (2012). Caveolin-1 reduces HIV-1 infectivity by restoration of HIV Nef mediated impairment of cholesterol efflux by apoA-I. Retrovirology.

[67] Zheng YH, Plemenitas A, Fielding CJ, Peterlin BM (2003). Nef increases the synthesis of and transports cholesterol to lipid rafts and HIV-1 progeny virions. Proc Natl Acad Sci U S A.

[68] Dyer MD, Murali TM, Sobral BW (2008). The landscape of human proteins interacting with viruses and other pathogens. PLoS Pathog.

[69] Chatr-Aryamontri A, Ceol A, Peluso D, Nardozza A, Panni S, Sacco F, Tinti M, Smolyar A, Castagnoli L, Vidal M, Cusick ME, Cesareni G (2009). VirusMINT: A viral protein interaction database. Nucleic Acids Res.

[70] Casella CR, Rapaport EL, Finkel TH (1999). Vpu increases susceptibility of human immunodeficiency virus type 1- infected cells to Fas killing. J Virol.

[71] Février M, Dorgham K, Rebollo A (2011). CD4 + T cell depletion in human immunodeficiency virus (HIV) infection: Role of apoptosis. Viruses.

[72] Garg H, Mohl J, Joshi A (2012). HIV-1 induced bystander apoptosis. Viruses.

[73] Hallstrom TC, Mori S, Nevins JR (2008). An E2F1-Dependent Gene Expression Program that Determines the Balance between Proliferation and Cell Death. Cancer Cell.

[74] O'Donnell KA, Wentzel EA, Zeller KI, Dang CV, Mendell JT (2005). c-Myc-regulated microRNAs modulate E2F1 expression. Nature.

[75] Jowett JBM, Planelles V, Poon B, Shah NP, Chen ML, Chen ISY (1995). The human immunodeficiency virus type 1 vpr gene arrests infected T cells in the G2 + M phase of the cell cycle. J Virol.

[76] Turelli P, Doucas V, Craig E, Mangeat B, Klages N, Evans R, Kalpana G, Trono D (2001). Cytoplasmic Recruitment of INI1 and PML on Incoming HIV Preintegration Complexes: Interference with Early Steps of Viral Replication. Mol Cell.

[77] Daniel R, Kao G, Taganov K, Greger JG, Favorova O, Merke G, Yen TJ, Katz RA, Skalka AM (2003). Evidence that the retroviral DNA integration process triggers an ATR-dependent DNA damage response. Proc Natl Acad Sci U S A.

[78] Claassen GF, Hann SR (1999). Myc-mediated transformation: The repression connection. Oncogene.

[79] Fan Y, Zou W, Green LA, Kim BO, He JJ (2011). Activation of Egr-1 expression in astrocytes by HIV-1 Tat: New insights into astrocyte-mediated Tat neurotoxicity. J Neuroimmune Pharmacol.

[80] Gersten M, Alirezaei M, Marcondes MCG, Flynn C, Ravasi T, Ideker T, Fox HS (2009). An integrated systems analysis implicates EGR1 downregulation in simian immunodeficiency virus encephalitis-induced neural dysfunction. J Neurosci.

[81] Kartvelishvili A, Lesner A, Szponar M, Simm M (2004). Microarray analysis of differentially expressed genes in cells resistant to HIV-1. Immunol Lett.

[82] Olivares I, Ballester A, Lombardia L, Dominguez O, López-Galíndez C (2009). Human immunodeficiency virus type 1 chronic infection is associated with different gene expression in MT-4, H9 and U937 cell lines. Virus Res.

[83] Wout AB V’t, Lehrman GK, Mikheeva SA, O'Keeffe GC, Katze MG, Bumgarner RE, Geiss GK, Mullins JI (2003). Cellular gene expression upon human immunodeficiency virus type 1 infection of CD4+-T-cell lines. J Virol.

[84] Van't Wout AB, Swain JV, Schindler M, Rao U, Pathmajeyan MS, Mullins JI, Kirchhoff F (2005). Nef induces multiple genes involved in cholesterol synthesis and uptake in human immunodeficiency virus type 1-infected T cells. J Virol.

[85] Koenig R, Zhou Y, Elleder D, Diamond TL, Bonamy GMC, Irelan JT, Chiang CY, Tu BP, De Jesus PD, Lilley CE, Seidel S, Opaluch AM, Caldwell JS, Weitzman MD, Kuhen KL, Bandyopadhyay S, Ideker T, Orth AP, Miraglia LJ, Bushman FD, Young JA, Chanda SK (2008). Global analysis of host-pathogen interactions that regulate early-stage HIV-1 replication. Cell.

[86] Zhou H, Xu M, Huang Q, Gates AT, Zhang XD, Castle JC, Stec E, Ferrer M, Strulovici B, Hazuda DJ, Espeseth AS (2008). Genome-Scale RNAi Screen for Host Factors Required for HIV Replication. Cell Host & Microbe.

[87] Yeung ML, Houzet L, Yedavalli VSRK, Jeang KT (2009). A genome-wide short hairpin RNA screening of Jurkat T-cells for human proteins contributing to productive HIV-1 replication. J Biol Chem.

[88] Pache L, König R, Chanda SK (2011). Identifying HIV-1 host cell factors by genome-scale RNAi screening. Methods.

[89] Parolini I, Sargiacomo M, Galbiati F, Rizzo G, Grignani F, Engelman JA, Okamoto T, Ikezu T, Scherer PE, Mora R, Rodriguez-Boulan E, Peschle C, Lisanti MP (1999). Expression of caveolin-1 is required for the transport of caveolin-2 to the plasma membrane. Retention of caveolin-2 at the level of the Golgi complex. J Biol Chem.

[90] Jäger S, Cimermancic P, Gulbahce N, Johnson JR, McGovern KE, Clarke SC, Shales M, Mercenne G, Pache L, Li K, Hernandez H, Jang GM, Roth SL, Akiva E, Marlett J, Stephens M, D'Orso I, Fernandes J, Fahey McMahon C, Oĝdonoghue AJ, Todorovic A, Morris JH, Maltby DA, Alber T, Cagney G, Bushman FD, Young JA, Chanda SK, Sundquist WI (2012). Global landscape of HIV-human protein complexes. Nature.

[91] Almond N, Jenkins A, Slade A, Heath A, Cranage M, Kitchin P (1992). Population sequence analysis of a simian immunodeficiency virus (32H reisolate of SIV(mac251): A virus stock used for international vaccine studies. AIDS Res Hum Retroviruses.

[92] Stahl-Hennig C, Dittmer U, Nisslein T, Petry H, Jurkiewicz E, Fuchs D, Wachter H, Mätz-Rensing K, Kuhn EM, Kaup FJ, Rud EW, Hunsmann G (1996). Rapid development of vaccine protection in macaques by live-attenuated simian immunodeficiency virus. J Gen Virol.

[93] Stahl-Hennig C, Steinman RM, Tenner-Racz K, Pope M, Stolte N, Mätz-Rensing K, Grobschupff G, Raschdorff B, Hunsmann G, Racz P (1999). Rapid infection of oral mucosal-associated lymphoid tissue with simian immunodeficiency virus. Science.

[94] Reed LJ, Muench HA (1938). Simple method of estimating fifty per cent endpoints. Am J Hyg.

[95] Negri DRM, Baroncelli S, Catone S, Comini A, Michelini Z, Maggiorella MT, Sernicola L, Crostarosa F, Belli R, Mancini MG, Farcomeni S, Fagrouch Z, Ciccozzi M, Boros S, Liljestrom P, Norley S, Heeney J, Titti F (2004). Protective efficacy of a multicomponent vector vaccine in cynomolgus monkeys after intrarectal simian immunodeficiency virus challenge. J Gen Virol.

[96] Goetze S, Huesemann Y, Baer A, Bode J (2003). Functional characterization of transgene integration patterns by halo fluorescence in situ hybridization: Electroporation versus retroviral infection. Biochemistry.

[97] Ma H, Zeng AP (2003). Reconstruction of metabolic networks from genome data and analysis of their global structure for various organisms. Bioinformatics.

[98] Castillo-Davis CI, Hartl DL (2003). GeneMerge - Post-genomic analysis, data mining, and hypothesis testing. Bioinformatics.

