# Supplementary figures and images for: Identification of molecular sub-networks associated with cell survival in a chronically SIVmac-infected human CD4+ T cell line
Source: Virol J. 2014 Aug 27;11:152. doi: 10.1186/1743-422X-11-152 (PMC4163169; doi:10.1186/1743-422X-11-152)

## Slide 1
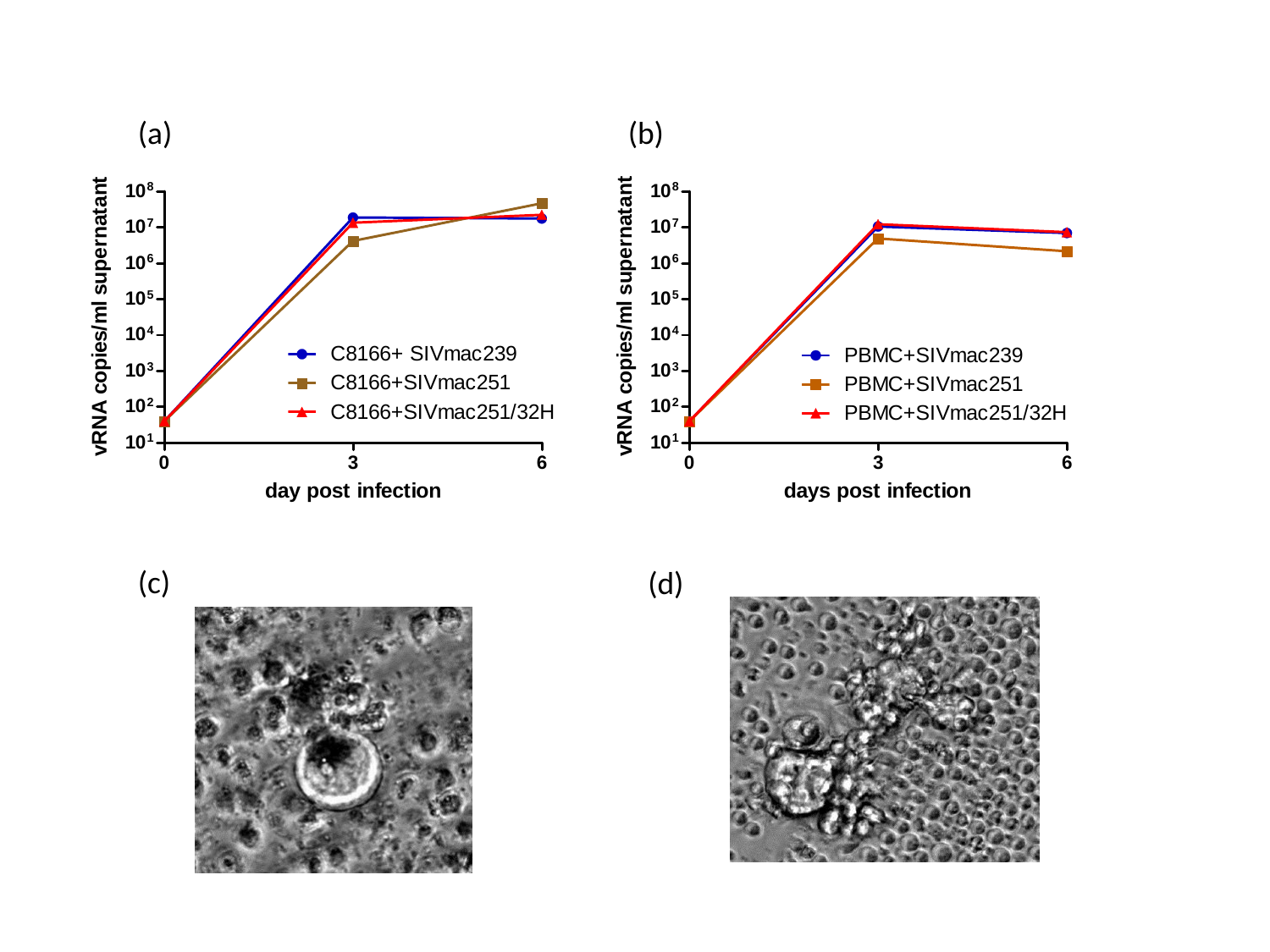

(a)
(b)
(c)
(d)

Supplement: Supplementary file 1 — Additional file 1: Figure S1: Virus produced by C8166-P cells (chronically SIV-infected cells) replicates similarly compared to highly pathogenic SIVmac strains and causes a lytic infection. Description: In vitro viral replication pattern of different SIV virus strains: (a) viral RNA copies in cell culture supernatant of in vitro infected C8166 cells, (b) viral RNA copies in cell culture supernatant of in vitro infected rhesus monkey peripheral blood mononuclear cells (PBMCs). Before infection, C8166 cells and rhesus PBMCs were washed. 2x10^6^ cells were re-suspended in 2 ml cell culture medium containing 50,000 TCID50 of SIVmac251/32H (supernatant of C8166-P), or 60,000 TCID50 SIVmac239 grown on C8166 cells or 30,000 TCID50 SIVmac251 grown on rhesus monkey lymphocytes. After an incubation step for 2 h at room temperature, cells were washed twice and cultured in 1 ml RPMI 1640 medium (PAN Biotech, Aidenbach, Germany) supplemented with 10% fetal calf serum (PAN Biotech, Aidenbach, Germany), 100 U/ml penicillin (PAN Biotech, Aidenbach, Germany) and 100 μg/ml streptomycin (PAN Biotech, Aidenbach, Germany; complete RPMI 1640). Before infection, frozen rhesus monkey PBMCs were thawed and stimulated with 2.5 μg ConA/ml overnight. Infection with different SIV strains was performed as described above. After infection, monkey PBMCs were cultured in RPMI 1640 complete medium supplemented with 100 U/ml recombinant human IL-2 (PeproTech, Hamburg, Germany). Viral RNA copies in cell culture supernatant were determined at day 3 and 6 post infection as described in Materials and Methods. Syncytia as evidence of lytic infection are observed after infection of C8166 cells (c) and PBMCs (d) with supernatant of C8166-P. (PPTX 241 KB) [file 12985_2013_2486_MOESM1_ESM.pptx]

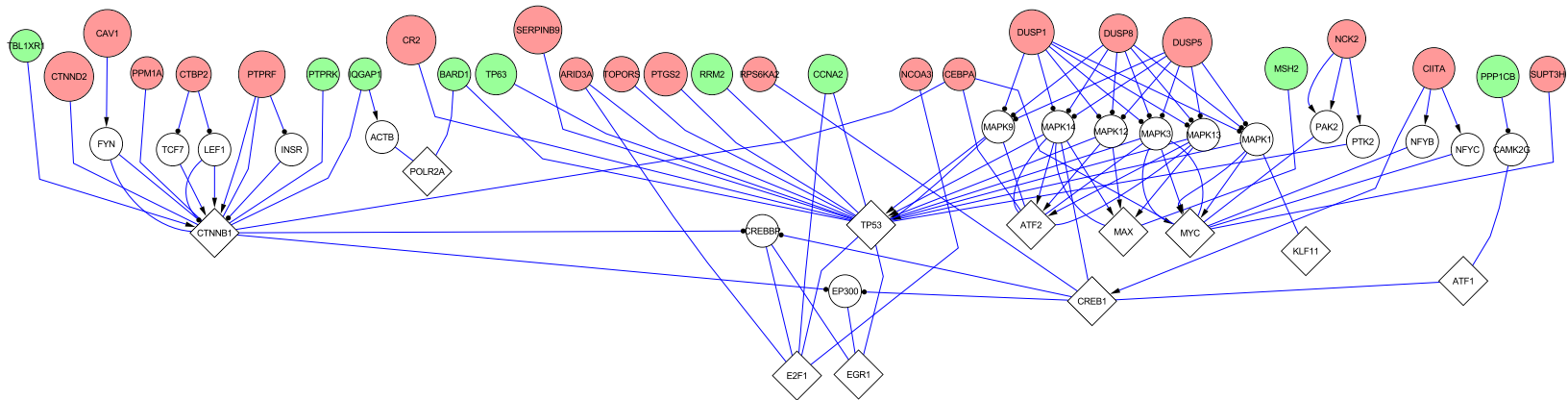

Supplement: Supplementary file 2 — Additional file 2: Figure S3: Significantly affected interactive molecular chains (IMCs) from chronically SIV-infected samples, which might modulate the activity of the ‘hidden’ key regulators. Different ‘hidden’ key regulators exhibiting no change in their RNA level are inferred by tracing the upstream interactive molecular network. Regulators are represented by diamonds. The black delta represents activation; the black circle represents inhibition; a simple line without a symbol represents an undirected relationship PPI of the two proteins. Red indicates significantly increased, green, significantly decreased. (PDF 60 KB) [file 12985_2013_2486_MOESM2_ESM.pdf]

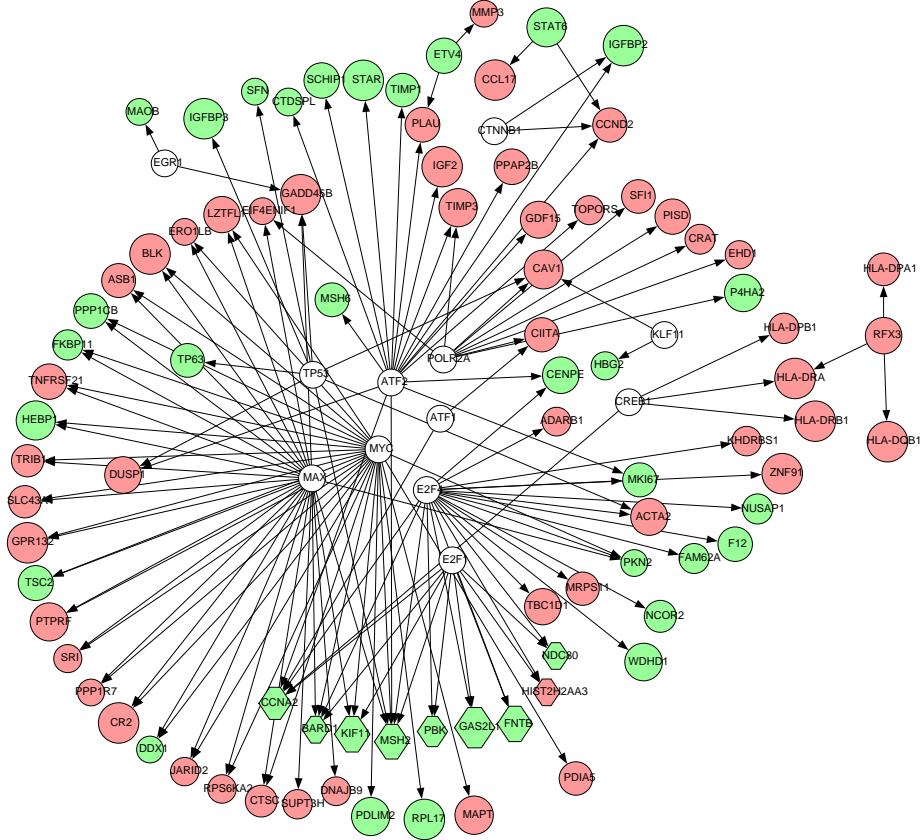

Supplement: Supplementary file 3 — Additional file 3: Figure S4: Parts of putative, non-differentially expressed key regulators and the affected transcription regulatory network identified by the ICF. The figure shows genes that are regulated at the RNA level in the chronically SIV- infected T cell line by non-differentially expressed (NDiff), putative key regulators. The direction of the arrow is from the regulator to the target gene. The target genes of E2F1 are indicated by hexagons. For a detailed discussion of the effects of P53, Myc, E2F and p53 on target genes and the cell, see text. The activity of transcription factor CREB is accompanied by the upregulation of members of the MHC class II family (HLA-D) and the downregulation of cyclin A 2 (CCNA2), a protein that activates certain kinases to promote cell cycle transitions. (PDF 30 KB) [file 12985_2013_2486_MOESM3_ESM.pdf]

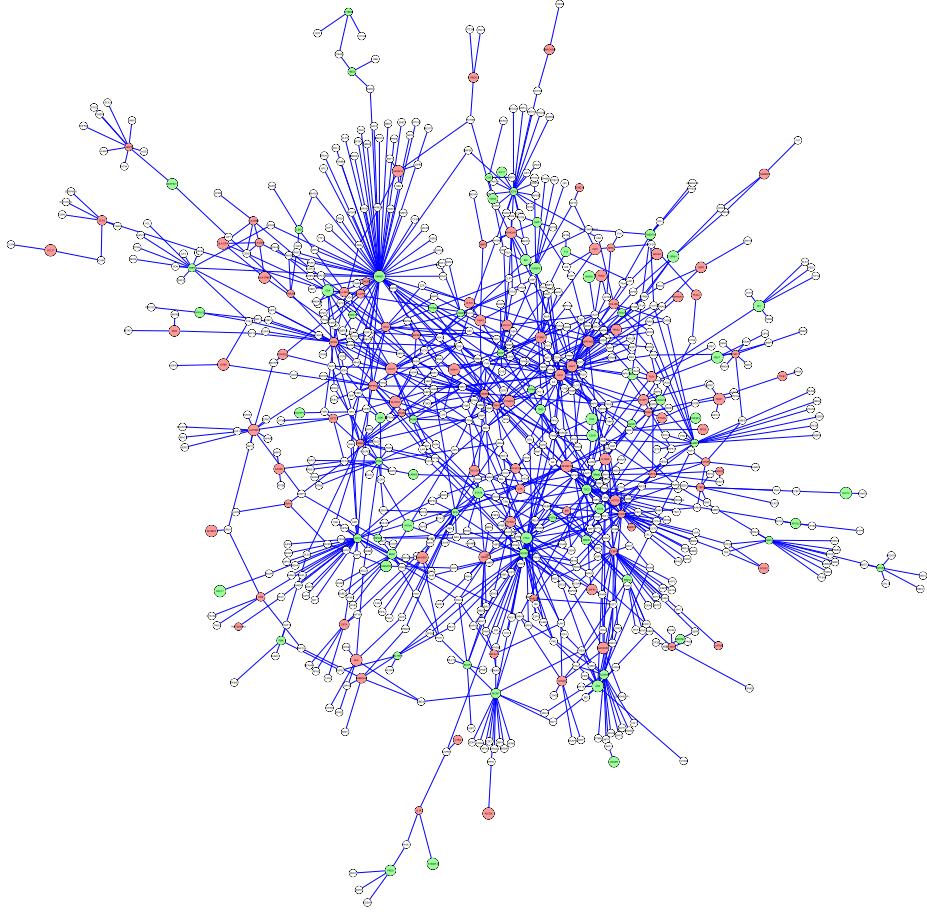

Supplement: Supplementary file 5 — Additional file 5: Figure S2: Protein-protein network of CD4+ T cells significantly perturbed only in the chronically SIV-infected cells identified by the ICF. Affected sub-networks in chronically infected C8166 cells are shown. Red colour, significantly increased; green, significantly decreased; white, non-differentially expressed (NDiff). (PDF 539 KB) [file 12985_2013_2486_MOESM5_ESM.pdf]
