# Supplementary material for: Identification of molecular sub-networks associated with cell survival in a chronically SIVmac-infected human CD4+ T cell line
Source: Virol J. 2014 Aug 27;11:152. doi: 10.1186/1743-422X-11-152 (PMC4163169; doi:10.1186/1743-422X-11-152)
Supplement: Supplementary file 4 — Additional file 4: Changes in gene expression in chronically and acutely SIV- infected C8166 T cells. The file lists up- and downregulated genes in chronically and acutely SIV-infected C8166 cells and the degree of regulation (changes in gene expression, log2 transformed from fold changes, versus mock-infected control cells). (DOCX 28 KB) [file 12985_2013_2486_MOESM4_ESM.docx]

**Additional file**

**Table A** **Significantly differentially expressed genes in chronic infection**.

| Entrez gene ID | average change in expression (fold, log 2) | Gene symbol |
| --- | --- | --- |
| 3514 | 5.9735 | IGKC |
| 1380 | 5.713 | CR2 |
| 57379 | 4.921 | AICDA |
| 7644 | 4.628 | ZNF91 |
| 55540 | 4.6155 | IL17RB |
| 3507 | 4.605 | IGHM |
| 7133 | 4.602 | TNFRSF1B |
| 9899 | 4.5875 | SV2B |
| 149111 | 4.1895 | CNIH3 |
| 22836 | 4.189 | RHOBTB3 |
| 10472 | 4.174 | ZNF238 |
| 6361 | 3.892 | CCL17 |
| 9500 | 3.85 | MAGED1 |
| 440871 | 3.6935 | LOC440871 |
| 23245 | 3.689 | ASTN2 |
| 753 | 3.6285 | C18orf1 |
| 919 | 3.546 | CD247 |
| 3119 | 3.3455 | HLA-DQB1 |
| 9215 | 3.2755 | LARGE |
| 3122 | 3.2165 | HLA-DRA |
| 29933 | 3.182 | GPR132 |
| 4481 | 3.1795 | MSR1 |
| 745 | 3.1615 | C11orf9 |
| 3123 | 3.0935 | HLA-DRB1 |
| 8728 | 3.0725 | ADAM19 |
| 54491 | 3.025 | FAM105A |
| 5996 | 3.0135 | RGS1 |
| 1501 | 3.0055 | CTNND2 |
| 5649 | 3.0015 | RELN |
| 3601 | 2.972 | IL15RA |
| 6549 | 2.9465 | SLC9A2 |
| 931 | 2.925 | MS4A1 |
| 4345 | 2.825 | CD200 |
| 3669 | 2.789 | ISG20 |
| 51029 | 2.731 | FAM152A |
| 3481 | 2.6235 | IGF2 |
| 9473 | 2.59 | C1orf38 |
| 640 | 2.5765 | BLK |
| 23677 | 2.5575 | SH3BP4 |
| 1847 | 2.555 | DUSP5 |
| 5799 | 2.5155 | PTPRN2 |
| 442039 | 2.4895 | LOC442039 |
| 29909 | 2.4875 | GPR171 |
| 11025 | 2.473 | LILRB3 |
| 8932 | 2.4455 | MBD2 |
| 4616 | 2.429 | GADD45B |
| 7136 | 2.425 | TNNI2 |
| 23108 | 2.3735 | GARNL4 |
| 283650 | 2.3345 | IGHA1 |
| 5272 | 2.3245 | SERPINB9 |
| 7805 | 2.3195 | LAPTM5 |
| 23779 | 2.2945 | ARHGAP8 |
| 25861 | 2.285 | DFNB31 |
| 25825 | 2.2805 | BACE2 |
| 857 | 2.268 | CAV1 |
| 4137 | 2.1915 | MAPT |
| 55647 | 2.185 | RAB20 |
| 7078 | 2.165 | TIMP3 |
| 5792 | 2.157 | PTPRF |
| 22795 | 2.1465 | NID2 |
| 4597 | 2.106 | MVD |
| 2570 | 2.097 | GABRR2 |
| 5553 | 2.0945 | PRG2 |
| 1268 | 2.0445 | CNR1 |
| 51174 | 2.0405 | TUBD1 |
| 860 | 2.0015 | RUNX2 |
| 59 | 1.989 | ACTA2 |
| 10667 | 1.985 | FARS2 |
| 9518 | 1.976 | GDF15 |
| 8869 | 1.9625 | ST3GAL5 |
| 1843 | 1.9475 | DUSP1 |
| 23705 | 1.9405 | CADM1 |
| 5078 | 1.922 | PAX4 |
| 5991 | 1.914 | RFX3 |
| 4651 | 1.9045 | MYO10 |
| 2517 | 1.9015 | FUCA1 |
| 55769 | 1.9005 | ZNF83 |
| 23406 | 1.899 | COTL1 |
| 80237 | 1.8965 | ELL3 |
| 54585 | 1.8915 | LZTFL1 |
| 23216 | 1.8825 | TBC1D1 |
| 54502 | 1.8765 | RBM47 |
| 7092 | 1.8695 | TLL1 |
| 2295 | 1.834 | FOXF2 |
| 6925 | 1.8315 | TCF4 |
| 23598 | 1.819 | PATZ1 |
| 53405 | 1.801 | CLIC5 |
| 51237 | 1.7965 | MZB1 |
| 8613 | 1.7885 | PPAP2B |
| 5743 | 1.776 | PTGS2 |
| 55332 | 1.77 | DRAM |
| 9148 | 1.7675 | NEURL |
| 27242 | 1.7605 | TNFRSF21 |
| 10765 | 1.7475 | JARID1B |
| 23761 | 1.747 | PISD |
| 10125 | 1.741 | RASGRP1 |
| 3418 | 1.728 | IDH2 |
| 3128 | 1.7265 | HLA-DRB6 |
| 65108 | 1.7115 | MARCKSL1 |
| 8623 | 1.711 | ASMTL |
| 9454 | 1.71 | HOMER3 |
| 79877 | 1.6845 | DCAKD |
| 5328 | 1.651 | PLAU |
| 9246 | 1.6205 | UBE2L6 |
| 115123 | 1.6015 | MARCH3 |
| 81552 | 1.6005 | ECOP |
| 8553 | 1.595 | BHLHB2 |
| 894 | 1.59 | CCND2 |
| 4261 | 1.589 | CIITA |
| 81618 | 1.5745 | ITM2C |
| 92815 | 1.5635 | HIST3H2A |
| 1236 | 1.561 | CCR7 |
| 51665 | 1.545 | ASB1 |
| 23645 | 1.5275 | PPP1R15A |
| 50626 | 1.51 | CYHR1 |
| 1991 | 1.4995 | ELA2 |
| 9814 | 1.4995 | SFI1 |
| 1645 | 1.494 | AKR1C1 |
| 10954 | 1.4915 | PDIA5 |
| 3635 | 1.491 | INPP5D |
| 64963 | 1.4855 | MRPS11 |
| 9263 | 1.474 | STK17A |
| 3113 | 1.46 | HLA-DPA1 |
| 4049 | 1.455 | LTA |
| 8501 | 1.442 | SLC43A1 |
| 10221 | 1.4385 | TRIB1 |
| 960 | 1.428 | CD44 |
| 56255 | 1.4225 | TXNDC13 |
| 1075 | 1.4215 | CTSC |
| 6367 | 1.418 | CCL22 |
| 9353 | 1.4095 | SLIT2 |
| 1850 | 1.3995 | DUSP8 |
| 8440 | 1.3985 | NCK2 |
| 4705 | 1.3775 | NDUFA10 |
| 55905 | 1.376 | ZNF313 |
| 1607 | 1.3665 | DGKB |
| 3115 | 1.3585 | HLA-DPB1 |
| 3855 | 1.357 | KRT7 |
| 1384 | 1.334 | CRAT |
| 3267 | 1.33 | HRB |
| 2852 | 1.319 | GPER |
| 10657 | 1.3185 | KHDRBS1 |
| 8445 | 1.3155 | DYRK2 |
| 3109 | 1.315 | HLA-DMB |
| 8464 | 1.313 | SUPT3H |
| 863 | 1.3025 | CBFA2T3 |
| 10210 | 1.3 | TOPORS |
| 7107 | 1.298 | GPR137B |
| 1488 | 1.2905 | CTBP2 |
| 9448 | 1.2865 | MAP4K4 |
| 104 | 1.285 | ADARB1 |
| 4189 | 1.285 | DNAJB9 |
| 6196 | 1.278 | RPS6KA2 |
| 3108 | 1.2755 | HLA-DMA |
| 25976 | 1.2725 | TIPARP |
| 3720 | 1.2685 | JARID2 |
| 10938 | 1.262 | EHD1 |
| 6717 | 1.258 | SRI |
| 3126 | 1.2405 | HLA-DRB4 |
| 10529 | 1.236 | NEBL |
| 6616 | 1.22 | SNAP25 |
| 1820 | 1.2195 | ARID3A |
| 90634 | 1.216 | N4BP2L1 |
| 655 | 1.21 | BMP7 |
| 4357 | 1.1995 | MPST |
| 4126 | 1.199 | MANBA |
| 2626 | 1.1975 | GATA4 |
| 9320 | 1.194 | TRIP12 |
| 3300 | 1.1915 | DNAJB2 |
| 56478 | 1.179 | EIF4ENIF1 |
| 2066 | 1.17 | ERBB4 |
| 3552 | 1.1645 | IL1A |
| 5510 | 1.156 | PPP1R7 |
| 5494 | 1.1505 | PPM1A |
| 25829 | 1.1475 | TMEM184B |
| 286527 | 1.1465 | TMSB15B |
| 50509 | 1.1425 | COL5A3 |
| 81619 | 1.139 | TSPAN14 |
| 4314 | 1.119 | MMP3 |
| 56605 | 1.112 | ERO1LB |
| 1050 | 1.102 | CEBPA |
| 3449 | 1.101 | IFNA16 |
| 5552 | 1.1005 | SRGN |
| 2798 | 1.1 | GNRHR |
| 8202 | 1.098 | NCOA3 |
| 1646 | 1.073 | AKR1C2 |
| 4162 | 1.0695 | MCAM |
| 8337 | 1.0665 | HIST2H2AA3 |
| 10107 | 1.0625 | TRIM10 |
| 1911 | 1.0405 | PHC1 |
| 10432 | -1.034 | RBM14 |
| 1639 | -1.0935 | DCTN1 |
| 79718 | -1.098 | TBL1XR1 |
| 1653 | -1.1185 | DDX1 |
| 7072 | -1.1205 | TIA1 |
| 4129 | -1.128 | MAOB |
| 2810 | -1.1385 | SFN |
| 26010 | -1.1395 | SPATS2L |
| 5796 | -1.159 | PTPRK |
| 8826 | -1.1625 | IQGAP1 |
| 57142 | -1.164 | RTN4 |
| 760 | -1.1695 | CA2 |
| 10403 | -1.1795 | NDC80 |
| 2526 | -1.186 | FUT4 |
| 27332 | -1.1965 | ZNF638 |
| 5701 | -1.205 | PSMC2 |
| 580 | -1.2085 | BARD1 |
| 1528 | -1.2165 | CYB5A |
| 1901 | -1.2415 | EDG1 |
| 10217 | -1.2425 | CTDSPL |
| 7468 | -1.2465 | WHSC1 |
| 9844 | -1.2485 | ELMO1 |
| 9801 | -1.2525 | MRPL19 |
| 7205 | -1.2545 | TRIP6 |
| 3048 | -1.2595 | HBG2 |
| 29990 | -1.262 | PILRB |
| 221061 | -1.291 | C10orf38 |
| 339 | -1.3 | APOBEC1 |
| 54880 | -1.3 | BCOR |
| 55179 | -1.3155 | FAIM |
| 7514 | -1.3375 | XPO1 |
| 23344 | -1.342 | FAM62A |
| 51203 | -1.344 | NUSAP1 |
| 51303 | -1.3495 | FKBP11 |
| 445815 | -1.3545 | PALM2-AKAP2 |
| 55872 | -1.358 | PBK |
| 57110 | -1.361 | HRASLS |
| 373156 | -1.361 | GSTK1 |
| 23244 | -1.386 | PDS5A |
| 1849 | -1.389 | DUSP7 |
| 26191 | -1.392 | PTPN22 |
| 8895 | -1.3965 | CPNE3 |
| 54892 | -1.411 | NCAPG2 |
| 54877 | -1.4235 | ZCCHC2 |
| 55056 | -1.4235 | FLJ10038 |
| 9612 | -1.4275 | NCOR2 |
| 890 | -1.4425 | CCNA2 |
| 391020 | -1.444 | LOC391020 |
| 7750 | -1.4605 | ZMYM2 |
| 155066 | -1.4655 | ATP6V0E2 |
| 195 | -1.476 | AHNAK |
| 7076 | -1.494 | TIMP1 |
| 23165 | -1.5105 | NUP205 |
| 8626 | -1.514 | TP63 |
| 6241 | -1.5155 | RRM2 |
| 84900 | -1.526 | TMEM118 |
| 2956 | -1.529 | MSH6 |
| 8609 | -1.541 | KLF7 |
| 6281 | -1.56 | S100A10 |
| 4288 | -1.5605 | MKI67 |
| 3911 | -1.5795 | LAMA5 |
| 81789 | -1.58 | TIGD6 |
| 4176 | -1.5975 | MCM7 |
| 5028 | -1.5975 | P2RY1 |
| 699 | -1.604 | BUB1 |
| 2161 | -1.6285 | F12 |
| 5500 | -1.654 | PPP1CB |
| 1830 | -1.6695 | DSG3 |
| 4436 | -1.671 | MSH2 |
| 244 | -1.687 | ANXA8L2 |
| 7249 | -1.703 | TSC2 |
| 486 | -1.738 | FXYD2 |
| 2118 | -1.742 | ETV4 |
| 5873 | -1.7425 | RAB27A |
| 285 | -1.746 | ANGPT2 |
| 928 | -1.749 | CD9 |
| 23596 | -1.7545 | OPN3 |
| 29970 | -1.761 | SCHIP1 |
| 23576 | -1.7625 | DDAH1 |
| 2342 | -1.771 | FNTB |
| 8906 | -1.7855 | AP1G2 |
| 3832 | -1.7955 | KIF11 |
| 55076 | -1.8155 | TMEM45A |
| 55668 | -1.826 | C14orf118 |
| 7292 | -1.8825 | TNFSF4 |
| 1062 | -1.9305 | CENPE |
| 56521 | -1.9495 | DNAJC12 |
| 26034 | -1.97 | PIP3-E |
| 5311 | -1.987 | PKD2 |
| 8942 | -1.994 | KYNU |
| 80765 | -2.002 | STARD5 |
| 307 | -2.006 | ANXA4 |
| 28639 | -2.0105 | TRBC1 |
| 5358 | -2.0265 | PLS3 |
| 8974 | -2.0525 | P4HA2 |
| 11169 | -2.0805 | WDHD1 |
| 23390 | -2.1225 | ZDHHC17 |
| 22824 | -2.1275 | HSPA4L |
| 3398 | -2.1545 | ID2 |
| 64236 | -2.163 | PDLIM2 |
| 28978 | -2.2025 | TMEM14A |
| 6038 | -2.248 | RNASE4 |
| 400451 | -2.2645 | FAM174B |
| 6778 | -2.2715 | STAT6 |
| 23075 | -2.281 | SWAP70 |
| 50865 | -2.3005 | HEBP1 |
| 23063 | -2.302 | WAPAL |
| 55471 | -2.327 | C2orf56 |
| 3397 | -2.361 | ID1 |
| 2911 | -2.3735 | GRM1 |
| 6284 | -2.4575 | S100A13 |
| 8519 | -2.466 | IFITM1 |
| 11278 | -2.484 | KLF12 |
| 55034 | -2.535 | MOCOS |
| 3486 | -2.538 | IGFBP3 |
| 6139 | -2.54 | RPL17 |
| 2274 | -2.5775 | FHL2 |
| 1466 | -2.603 | CSRP2 |
| 768 | -2.889 | CA9 |
| 283 | -2.9025 | ANG |
| 545 | -2.905 | ATR |
| 130872 | -2.9445 | AHSA2 |
| 9935 | -2.946 | MAFB |
| 2014 | -2.9665 | EMP3 |
| 2529 | -3.0095 | FUT7 |
| 920 | -3.018 | CD4 |
| 4291 | -3.1525 | MLF1 |
| 3241 | -3.2745 | HPCAL1 |
| 4900 | -3.4345 | NRGN |
| 10351 | -3.544 | ABCA8 |
| 1005 | -3.567 | CDH7 |
| 9235 | -3.709 | IL32 |
| 6770 | -3.8325 | STAR |
| 10634 | -4.4835 | GAS2L1 |
| 366 | -4.5215 | AQP9 |
| 3485 | -4.593 | IGFBP2 |
| 4086 | -4.6635 | SMAD1 |
| 9447 | -5.0895 | AIM2 |
| 6322 | -5.912 | SCML1 |

**Table B** **Significantly differentially expressed genes in acute infection.**

| Entrez gene ID | average change in expression (fold, log2) | Gene symbol |
| --- | --- | --- |
| 5238 | 3.312 | PGM3 |
| 55054 | 3.068 | ATG16L1 |
| 2690 | 2.8905 | GHR |
| 26692 | 2.8325 | OR2W1 |
| 10272 | 2.478 | FSTL3 |
| 51341 | 2.4 | ZBTB7A |
| 139538 | 2.376 | VENTXP1 |
| 23365 | 2.16 | ARHGEF12 |
| 6367 | 2.0535 | CCL22 |
| 4089 | 2.039 | SMAD4 |
| 2615 | 1.632 | LRRC32 |
| 4189 | 1.5475 | DNAJB9 |
| 1847 | 1.524 | DUSP5 |
| 54516 | 1.4435 | MTRF1L |
| 23580 | 1.3485 | CDC42EP4 |
| 23353 | 1.345 | UNC84A |
| 51279 | 1.329 | C1RL |
| 4616 | 1.3095 | GADD45B |
| 10938 | 1.275 | EHD1 |
| 11176 | 1.1945 | BAZ2A |
| 9414 | 1.1845 | TJP2 |
| 2065 | 1.062 | ERBB3 |
| 5142 | -1.1805 | PDE4B |
| 3398 | -1.3375 | ID2 |
| 6954 | -1.4585 | TCP11 |
| 55139 | -1.623 | ANKZF1 |
| 81790 | -1.7085 | RNF170 |
| 1258 | -1.7505 | CNGB1 |
| 57194 | -2.165 | ATP10A |
| 23033 | -2.872 | DOPEY1 |
| 1825 | -2.9685 | DSC3 |
